# A Refined View of Airway Microbiome in Chronic Obstructive Pulmonary Disease at Species and Strain-Levels

**DOI:** 10.3389/fmicb.2020.01758

**Published:** 2020-07-30

**Authors:** Zhang Wang, Haiyue Liu, Fengyan Wang, Yuqiong Yang, Xiaojuan Wang, Boxuan Chen, Martin R. Stampfli, Hongwei Zhou, Wensheng Shu, Christopher E. Brightling, Zhenyu Liang, Rongchang Chen

**Affiliations:** ^1^Institute of Ecological Science, School of Life Sciences, South China Normal University, Guangzhou, China; ^2^State Key Laboratory of Organ Failure Research, Microbiome Medicine Center, Division of Laboratory Medicine, Zhujiang Hospital, Southern Medical University, Guangzhou, China; ^3^State Key Laboratory of Respiratory Disease, National Clinical Research Center for Respiratory Disease, Guangzhou Institute of Respiratory Health, The First Affiliated Hospital of Guangzhou Medical University, Guangzhou, China; ^4^Department of Medicine, Firestone Institute for Respiratory Health at St. Joseph’s Healthcare, McMaster University, Hamilton, ON, Canada; ^5^Institute for Lung Health, NIHR Leicester Biomedical Research Centre, Department of Respiratory Sciences, University of Leicester, Leicester, United Kingdom; ^6^Pulmonary and Critical Care Department, Shenzhen Institute of Respiratory Diseases, Shenzhen People’s Hospital, Shenzhen, China

**Keywords:** airway microbiome, COPD, full-length 16S sequencing, PacBio, airway inflammation

## Abstract

Little is known about the underlying airway microbiome diversity in chronic obstructive pulmonary disease (COPD) at in-depth taxonomic levels. Here we present the first insights on the COPD airway microbiome at species and strain-levels. The full-length 16S rRNA gene was characterized from sputum in 98 COPD patients and 27 age-matched healthy controls, using the Pacific Biosciences sequencing platform. Individual species within the same genus exhibited reciprocal relationships with COPD and disease severity. Species dominant in health can be taken over by another species within the same genus but with potentially increasing pathogenicity in severe COPD patients. *Ralstonia mannitolilytica*, an opportunistic pathogen, was significantly increased in frequent exacerbators (fold-change = 4.94, FDR *P* = 0.005). There were distinct patterns of interaction between bacterial species and host inflammatory mediators according to neutrophilic or eosinophilic inflammations, two major airway inflammatory phenotypes in COPD. *Haemophilus influenza*e, *Moraxella catarrhalis, Pseudomonas aeruginosa*, and *Neisseria meningitidis* were associated with enhanced Th1, Th17 and pro-inflammatory mediators, while a group of seven species including *Tropheryma whipplei* were specifically associated with Th2 mediators related to eosinophilia. We developed an automated pipeline to assign strain-level taxonomy leveraging bacterial intra-genomic 16S allele frequency. Using this pipeline we further resolved three non-typeable *H. influenzae* strains PittEE, PittGG and 86-028NP with reasonable precision and uncovered strain-level variation related to airway inflammation. In particular, 86-028NP and PittGG strains exhibited inverse associations with Th2 chemokines CCL17 and CCL13, suggesting their abundances may inversely predict eosinophilic inflammation. A systematic comparison of 16S hypervariable regions indicated V1V3 instead of the commonly used V4 region was the best surrogate for airway microbiome. The full-length 16S data augmented the power of functional inference, which slightly better recapitulated the actual metagenomes. This led to the unique identification of butyrate-producing and nitrate reduction pathways as depleted in COPD. Our analysis uncovered finer-scale airway microbial diversity that was previously underappreciated, thus enabled a refined view of the airway microbiome in COPD.

## Introduction

Chronic obstructive pulmonary disease (COPD) is a heterogeneous lung disease characterized by airflow obstruction and persistent airway inflammation. The respiratory microbiome in COPD has been well studied in the last decade. The airway microbiome differs between health and COPD (Pragman et al., 2012; Sze et al., 2012; Zakharkina et al., 2013; Einarsson et al., 2016), shifts during acute exacerbations (Huang et al., 2014; Wang et al., 2016, 2018), associates with airway inflammation (Wang et al., 2016; Wang Z. et al., 2019) and predicts 1-year mortality of hospitalized exacerbation patients (Leitao Filho et al., 2018), all suggesting the implication of airway microbiome in COPD pathophysiology. Despite advances, the role of airway microbiome in COPD remains incompletely understood. An important knowledge gap is that our current view of airway microbiome is limited at most to its composition at bacterial genus-level, due to insufficient resolution of one or few hypervariable regions of 16S rRNA gene being sequenced in essentially all previous amplicon sequencing-based studies. In these studies, certain bacterial genera were often reported to be altered as a whole in disease and in relation to airway inflammation (Wang et al., 2016, 2018). However, from an ecological perspective, members of microbial community do not necessarily function according to their taxonomic groups, instead diversified species can act in the form of ecological “guilds” that co-adapt to altered environment (Zander, 2001; Zhao et al., 2018). Therefore, the aggregated genus-level associations can often be spurious or even misleading due to violation of basic ecological concepts. The inadequate depth of taxonomic profiling limits not only the accuracy of ecological inferences but also the ability to identify key bacterial species to use in follow-up experimental studies.

The recently advanced ‘third-generation’ sequencing technologies such as Pacific Biosciences (PacBio) and Nanopore is increasingly applied to microbiome studies (Goodwin et al., 2016; Levy and Myers, 2016). By generating long reads that extend tens of thousands of nucleotides, they offer the promise of increased taxonomic resolution by sequencing the full-length of 16S rRNA gene, thus serve as an attractive alternative for in-depth microbial taxonomic profiling (Wagner et al., 2016). In these applications, the 16S amplicon is circularized and read through multiple passes before circular consensus sequences (CCS) are generated, which greatly reduced the initial high error rate (∼10%) of the long-read sequencing to that comparable to short-read sequencing (∼0.5%) (Jiao et al., 2013; Hebert et al., 2018). Recent development of sophisticated denoising algorithms further enable accurate bacterial species identification at single-nucleotide resolution with near-zero error rate (Callahan et al., 2019). In some situations, strain-level identity can be further resolved utilizing information on the full complement of 16S rRNA gene alleles in bacterial genomes (Callahan et al., 2019; Johnson et al., 2019).

Here, we present the first analysis of the airway microbiome in COPD at species-level using PacBio sequencing. We also developed an automated pipeline to further resolve strain-level identity when possible. Our results uncovered additional diversity and heterogeneity in the airway microbiome at species-level and below, which was associated with patient clinical features and airway inflammation.

## Materials and Methods

### Patient Inclusion and Exclusion Criteria

Sputum samples of 98 stable COPD patients and 27 age-matched healthy controls were collected in the First Affiliated Hospital of Guangzhou Medical University. The study was approved and supervised by the ethics committee of the First Affiliated Hospital of Guangzhou Medical University (reference number: No. 2017-22) and was registered in www.clinicaltrials.gov (NCT 03240315). All COPD patients met the diagnostic criteria according to GOLD guideline and were also assessed for symptoms and exacerbation frequency (Table 1). GOLD classification for disease severity (stage I-IV) was assigned to COPD patients based on pulmonary function test. For COPD patients, the inclusion criteria were: (1) age > 40 years; and (2) confirmed diagnosis of COPD according to the GOLD guideline (post-bronchodilator forced expiratory volume in 1s [FEV1]/forced vital capacity [FVC] ratio < 0.7). The exclusion criteria were: (1) physician-diagnosis of asthma or significant respiratory disease other than COPD; (2) COPD exacerbation within 4 weeks of enrollment; (3) history of lung surgery and tuberculosis; (4) diagnosis of cancer; (5) blood transfusion within 4 weeks of enrollment; (6) diagnosis of autoimmune diseases; (7) enrollment in a blinded drug trial; and (8) short-term antibiotic usage within 4 weeks of enrollment. Informed consent was obtained from all patients.

**TABLE 1:** Major demographic and clinical characteristics of subjects.

**Demographic and clinical features**	**Healthy (*n* = 27)**	**COPD (*n* = 98)**	***P*-value**
Age	65.4 (10.8)	66.2 (8.9)	0.67
Gender, n(M/F)	23/4	89/9	0.48
Current smoking, n(Y/N)	9/18	85/13	1.0e−4***
GOLD (1/2/3/4)	NA	24/33/32/9	NA
New GOLD (a/b/c/d)^$^	NA	39/38/4/17	NA
Frequent exacerbator (Y/N) ^$$^	NA	20/78	NA
ICS usage (Y/N)	NA	58/40	NA
Long-term antibiotics (Y/N)	0/27	1/97	0.60
pre-FEV_1_ (L)	2.8 ± 0.1	1.5 ± 0.1	5.1e−10***
pre-FVC (L)	3.4 ± 0.2	2.9 ± 0.1	0.01**
pre-FEV_1_ (%)	100.0 ± 2.6	56.1 ± 2.7	1.0e−10***
pre-FEV_1_/FVC	0.81 ± 0.01	0.49 ± 0.02	2.5e−13***
post-FEV_1_ (L)	NA	1.6 ± 0.7	NA
post-FVC (L)	NA	3.1 ± 0.8	NA
post-FEV_1_ (%)	NA	59.6 ± 2.6	NA
post-FEV_1_/FVC	NA	0.5 ± 0.1	NA
CAT score	NA	4.0 ± 0.6	NA
mMRC	NA	0.4 ± 0.1	NA
Total sputum cells (cells × 10^9^/L)	NA	20.4 ± 2.8	NA
Sputum neutrophils (%)	NA	86.2 ± 1.2	NA
Sputum eosinophils (%)	NA	5.3 ± 0.7	NA
Sputum lymphocyte (%)	NA	0.7 ± 0.1	NA
Sputum monocyte (%)	NA	7.9 ± 1.1	NA
Blood neutrophils (cells × 10^9^/L)	NA	3.9 ± 0.1	NA
Blood eosinophils (cells × 10^9^/L)	NA	0.3 ± 0.0	NA
Blood lymphocyte (cells × 10^9^/L)	NA	1.8 ± 0.1	NA
Blood monocyte (cells × 10^9^/L)	NA	0.5 ± 0.1	NA

Continuous data are present as mean (range) or mean ± SEM. *P*-value was calculated using Fisher exact test for categorical variables and using Wilcoxon rank-sum test for continuous variables. ***P < 0.001; **P < 0.01; *P < 0.05 ICS: inhaled corticosteroids; FEV_1_: forced expiratory volume in 1 s; FVC: forced vital capacity, CAT: COPD Assessment Test, mMRC: modified Medical Research Council. ^$^The new GOLD classification based on mMRC, CAT and exacerbation frequency (2019) [Global Initiative for Chronic Obstructive Lung Disease (GOLD), 2019]. ^$$^The frequent exacerbator was defined as exacerbation event > = 2/last year.

### Quality Control of Sputum Samples

Induced sputum were obtained for all subjects and quality-controlled upon collection. Briefly, sputum plugs which contained the most viscous material were picked up in a petri dish and isolated from saliva. The selected sputum plugs were prepared for cytology by dilution with 0.1% dithiothreitol (DTT) solution and filtered through 48 μm nylon-mesh filter according to standardized sputum processing protocol (Bafadhel et al., 2012). The numbers of total cells, squamous epithelial cells and leukocytes were counted and recorded. Sputum specimens with squamous epithelial cells: leukocytes < 1:2.5 were considered unlikely to be contaminated with oropharyngeal flora and acceptable for downstream experiments (Murray and Washington, 1975; Roson et al., 2000).

### Sputum Inflammatory Mediators

A panel of 47 sputum mediators (BLC, Eotaxin, Eotaxin-2, CXCL11, CXCL10, CCL2, CCL3, CCL4, CCL5, CCL13, CCL17, G-CSF, GM-CSF, I-309, ICAM-1, IFNg, IL-1a, IL-1ra, IL-1b, IL-2, IL-4, IL-5, IL-6, IL-6R, IL-7, IL-8, IL-10, IL-12p40, IL-12p70, IL-13, IL-15, IL-16, IL-17, IL-21, MCSF, MIG, MIP-1d, MMP-8, MMP-9, PDGF-AB, Procalcitonin, TNFa, TNFb, TNFRI, TNFRII, TIMP-1, TIMP-2) were measured in a subset of 59 patients with available sputum samples using custom antibody microarray (Human Cytokine Antibody Microarray slides; RayBiotech Inc., Norcross, GA, United States) (Wang F. et al., 2019).

### The Full-Length 16S Sequencing and Analysis

Bacterial genomic DNA was extracted from selected sputum plugs using Qiagen DNA Mini kit. Negative controls for extraction (no sputum) and PCR amplification (no DNA template, ddH2O only) were included and sequenced together with all samples. The full-length (V1V9) bacterial 16S rRNA gene sequences were amplified using barcoded 27F (AGRGTTYGATYMTGGCTCAG) and 1492R (RGYTA CCTTGTTACGACTT) primers. Library construction was performed using Pacific Biosciences (PacBio) SMRTbell^TM^ Template Prep Kit V1 on normalized pooled PCR products, and was sequenced using PacBio Sequel platform.

Circular consensus sequences reads were generated using the *ccs* application in SMRTLink 5.1 with minPasses = 5 and minPredictedAccuracy = 0.90. The demultiplexed CCS were analyzed using DADA2 v1.12.1 customized for the PacBio full-length 16S sequencing data (Callahan et al., 2016, 2019). Briefly, primers were removed from the CCS sequences using the *removePrimers* function. Sequences without primer matches were discarded. The remaining CCS sequences were filtered using the *filterAndTrim* function with parameters: minLen = 1000, maxLen = 1600, maxN = 0, maxEE = 2, and minQ = 3. Sequences were dereplicated and used for learning the dataset-specific error model using the *learnErrors* function with parameter errorEstimationFunction = dada2:PacBioErrfun. Sequences were denoised using the error model and Amplicon Sequence Variants (ASVs) were identified using the *dada* function. Taxonomy up to the genus-level was assigned using the *assignTaxonomy* function based on the silva_nr_v128_train_set sequence database. ASVs were further assigned to species if they had exact unambiguous sequence match to species in the silva_species_assignment_v132 sequence database using the *addSpecies* function^1^. For the remaining ASVs, an additional step of BLASTn search was performed against the local NCBI nt database to make possible species-level calls based on exact unambiguous sequence match. Chimera sequences were removed using the *removeBimeraDenovo* function.

For comparison with the DADA2 approach, we performed two different clustering-based analyses that were previously used for full-length 16S amplicon sequences. First, we used the MCSMRT pipeline developed by Earl et al. for PacBio full-length 16S sequences (−*e* = 2), which clustered sequences into 97% operational taxonomic units (OTUs) and assigned taxonomy using a utax classifier on a custom-built full-length 16S database (Earl et al., 2018). Second, we performed a *de novo* clustering using the quality-filtered CCS reads into OTUs at 99% sequence identity using USEARCH (v11.0.667) (Edgar, 2010), according to Johnson et al. (2019). A custom Naïve Bayes classifier was trained on the Greengenes 13_8 99% OTUs using full-length 16S sequences obtained with 27F and 1492R primers, and applied to assign taxonomy for the representative sequence of each OTU using QIIME2 (Bolyen et al., 2018).

For downstream analyses, when analyzing microbiomes at the community level (i.e., alpha/beta diversity, functional inference), all ASVs were included irrespective of their taxonomic assignments. For differential analysis and correlation analyses with host mediators, we only focused on ASVs with species-level assignment, in order to identify species-level markers that were biologically interpretable. The ASV profile was rarefied to 3,119 reads per sample (Supplementary Figure S1).

### Metagenomic Sequencing and Analysis

To assess the precision of functional inference based on full-length 16S data, we randomly selected sputum samples from 10 healthy controls and 10 COPD patients for shotgun metagenomic sequencing as benchmark. Shotgun metagenomic library was prepared using the extracted genomic DNA and sequenced using Illumina NovaSeq platform. Quality filtering of the metagenomic reads was performed using Cutadapt v1.18 (–*q* = 20) (Marcel, 2011). Host reads were removed by mapping the quality-filtered reads to human reference genome hg38 using bowtie2 (Langmead and Salzberg, 2012). The remaining reads were subject to functional profiling using HUMANn2 (Franzosa et al., 2018). An average of 1.3 million reads per sample can be putatively assigned to KO genes (range 188,636–6,882,594 reads). The HUMANn2 profile was downsized to 188,636 reads per sample only when performing correlation analysis on gene features across all samples, to eliminate variations influenced by sequencing depth. The raw Fastq data of this study has been deposited in the Chinese National Gene Bank (CNGB) Nucleotide Sequence Archive (CNSA) under accession code CNP0000837.

### Strain-Level Identification Pipeline

Callahan et al. (2019) described a method for strain-level identification by hand using full-length 16S data leveraging the full complement of 16S rRNA alleles in bacterial genomes. In principle, a strain can be confidently assigned if all intra-genomic 16S sequence variants of that strain are recovered in integral ratios according to its genuine allelic variants. In extension to this approach, we designed an automated pipeline to assign strain-level ASV bins in four steps below. (1) All copies of 16S sequences were retrieved from 14,062 complete bacterial genomes in Genbank (assessed May 3, 2019) and the intra-genomic 16S copy number ratios were calculated. (2) All species-level ASVs were BLASTn-searched against the 16S database in step 1. ASVs with exact match to the same bacterial genome were assigned to the same initial bins. (3) The ASVs within each initial bin were subject to pairwise correlation, to generate refined bins by identifying ASVs with co-occurrence pattern (Pearson’s *R* > 0.7). (4) For each refined bin, the copy number ratio of ASVs were determined based on linear regression slope, and reconciled with the genuine copy number ratio of the 16S alleles in the corresponding bacterial genomes. The ASVs in integral copy number ratio with the genuine ratio (± 0.3) were retained in the final bins and assigned with strain-level taxonomy. The non-unique BLASTn matches (i.e., multiple genome-bins for the same set of ASVs) were resolved in this step using the genuine copy number ratio, when possible. The genomic abundances of each strain in each sample was calculated as the average abundances of ASVs normalized by their copy numbers in the genome. The curated intra-genomic 16S copy number ratio for all complete bacterial genomes and the code for the pipeline are available at GitHub^2^.

### Statistical Analysis

Differential microbiome features between COPD and healthy controls were identified using a linear discriminant analysis (LDA) effect size (LEfSe) method with a threshold of logarithmic LDA score 2.0 (Segata et al., 2011). Random forest analysis was performed using genus and species-level microbiome features selected by LEfSe (LDA > 2.0) using Weka 3.8 with 7-fold cross-validation (Frank et al., 2016). Area under receiver operative characteristic curve (AUC) were assessed to evaluate the performance of the random forest models. Statistical comparison of AUCs was performed using pROC package in R (Robin et al., 2011). Co-occurrence analysis of microbiome was performed using SparCC (Friedman and Alm, 2012). Functional inference of microbiome was performed using PICRUSt2 (Douglas et al., 2020). The false discovery rate (FDR) method was used to adjust *P*-values.

To identify microbiome-mediator associations independent of patient demographic factors, we performed a residualized correlation analysis (Lloyd-Price et al., 2019). All microbiome features and the 47 sputum mediators were first residualized using a general linear model adjusting for patient demographic covariates including age, gender, smoking history, exacerbation frequency and ICS usage. An all-against all correlation analysis was performed on the residues of microbiome features and sputum mediators using HAllA (Hierarchical All-against-All association testing) (Lloyd-Price et al., 2019), a computational tool to identify pairs of statistically significant associations in a hierarchical manner (FDR *P* < 0.05). A two-way hierarchical clustering analysis was performed on the microbiome-mediator correlation matrix using Ward’s method, in which microbiome features and mediators were both clustered. The mediators were clustered into three groups based on dendrogram and termed based on their inflammatory classes (Group 1: Th2-related, Group 2: Th1/Th17/Pro-inflammatory-related, Group 3: Others). The microbiome features were clustered into four groups based on dendrogram. The four microbiome groups were termed according to their association patterns with the three groups of mediators (“Pro-inflammatory,” “Neutrophilic,” “Eosinophilic,” and “Anti-inflammatory”). The “Neutrophilic” microbiome group had specific association pattern with Th1/Th17/Pro-inflammatory mediators. The “Eosinophilic” microbiome group had specific association pattern with Th2 mediators. The “Pro-inflammatory” and “Anti-inflammatory” microbiome groups had overall consistent positive or negative association pattern with the broad panel of inflammatory mediators. Patients were further stratified into eosinophilic-high and eosinophilic-low subgroups based on established criteria on sputum eosinophilic percentage (eosinophilic-high: sputum eosinophil > = 3%, eosinophilic-low: sputum eosinophil < 3%) (Bafadhel et al., 2011).

Individual partitions of 16S rRNA gene sequences were generated by truncating different hypervariable regions (V1V2, V1V3, V2, V3, V3V4, V3V5, V4, V6V8, and V6V9) from the full-length 16S sequences according to established primer sets using Cutadapt v2.6 (Marcel, 2011). The same DADA2 analysis as described above was performed on each individual partition. Mantel test was performed to assess the similarity in compositional and functional profiles generated using the full-length 16S sequences as well as using individual sub-regions.

### Quantitative PCR Assays

To validate our results for species identification and quantification, quantitative PCR assays were performed on a subset of 87 subjects with sufficient sputum genomic DNA (76 COPD patients and 11 controls). We designed species-specific primers for *Haemophilus influenzae, Haemophilus parainfluenzae*, and *Ralstonia mannitolilytica.* Species-specific genes were first identified using a BLASTp search of all protein-coding genes from each species against an in-house database of protein-coding genes from 2,764 complete bacterial genomes, to identify genes present in at least 90% of genomes of that species but absent in all other bacterial genomes. Primers were designed on the species-specific genes using Primer 3^3^ and were checked for primer-dimer and binding specificity to the bacterial genomes. We also identified strain-specific protein coding genes for *Haemophilus influenzae* strains PittEE, PittGG and 86-028NP using similar strategies as above, and designed primers based on these genes. The primer sequences for *Trophyrema whipplei* were adopted from the study of Lozupone et al. (2013), based on the *hsp65* gene sequence. To make standard curves for absolute quantification, an initial qPCR was performed on one of the sputum samples with sufficient quantity to amplify the PCR products using all primers. The PCR products were verified by Sanger sequencing and subject to standard *Escherichia coli* transformation procedure to obtain DNA templates with absolute copy numbers. The primers and their targeting genes for each species and sub-species were listed in Supplementary Table S1.

For validation of functional inference, we performed qPCR assays on butyryl-CoA:acetate CoA-transferase gene (EC:2.8.3.8) using the validated broad-spectrum primers reported by Vital et al. (2013) (but_3F: GHATYGGIGSTA TGCC, but_3R: AAGTCWAAYTGWCCRCC). The universal marker *rpoB* gene was used as the internal control in the qPCR assays (F: GGYTWYGAAGTNCGHGACGTDCA, R: TGACGYTGCATGTTBGMRCCCATMA). The fold change of the *but* gene between COPD and healthy controls was calculated using the 2^−ΔΔCt^ approach.

All qPCR assays were performed using 96-well MicroAmp Fast Optical 96-Well Reaction Plate on the Applied Biosystems StepOnePlus^TM^ Real-Time System. The 20 μl reaction mixture contained 10 μl of SYBR^®^ Select Master Mix (2×), 6 μl of microbial-free water, 2 μl DNA templates, and 1 μl forward and reverse primer each. The following cycling parameters were used: initial cycle of 95°C for 10 min; 40 cycles of 95°C for 15 s; 60°C for 1 min. All qPCR templates were run in duplicate. For standard curve calculation, each plate run included a decimal serial dilution of the corresponding double-stranded DNA templates as obtained above from 1E8 to 1E3 copies per μl. For qPCR of the functional gene (EC:2.8.3.8) using broad-spectrum primers, a high MgCl_2_ concentrations of 3mM was used and thermocycling was performed as follows: 95°C for 2 min; 95°C for 45 s, 54°C for 45 s, 72°C for 45 s (× 40); 10 min at 72°C, according to the original publication (Vital et al., 2013).

## Results

### Overview of the Species-Level Airway Microbiome Profile

**FIGURE 1:**
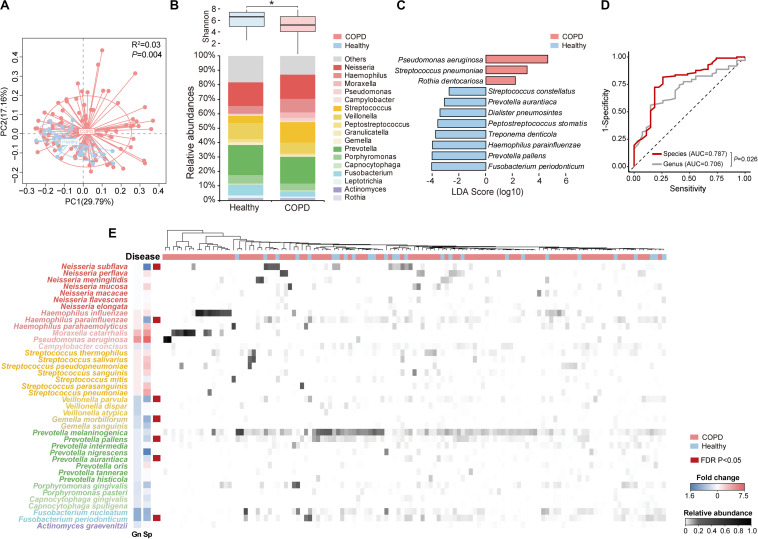
The overview of species-level profile of the airway microbiome in COPD patients and healthy controls. **(A)** Principal coordinate analysis based on weighted UniFrac distance on sputum samples from 98 COPD patients and 27 healthy controls. **(B)** The Shannon diversity and relative abundances of major genera (relative abundance > 0.005) in COPD patients and healthy controls. **(C)** The 11 top discriminatory species-level taxa between COPD and controls as identified from LEfSe analysis (LDA > 2.0). **(D)** The receiver operating characteristic curves for the Random Forest analyses using the 11 species-level and 9 genus-level discriminatory taxa (LDA > 2.0) to segregate COPD patients from controls. **(E)** The heatmap for the species-level microbiome profile. The major species-level taxa (relative abundance > 0.001) within each genus in panel **(B)** were shown. The fold change of each species (Sp) and its corresponding genus (Gn) in COPD patients versus controls were shown beside the taxonomy. * Wilcoxon test, *P* < 0.05.

A total of 1,317,570 high-quality CCS reads were obtained for 98 stable COPD patients and 27 healthy controls (Table 1). The average number of passes on the 16S gene was 34.9 for all CCS reads (5-270 passes), equivalent to a low error rate of ∼0.48% based on previous sequencing runs on a mock community (Johnson et al., 2019). A total of 2,868 non-singleton ASVs were identified using the DADA2 approach. Seven ASVs were present in reagent controls totaling 106 reads and were subtracted from the samples (Supplementary Table S2). At the community level, there were significant shifts in COPD patients versus controls (Figures 1A,B, Adonis, *P* = 0.004). There were no significant community shifts between smokers and non-smokers within COPD patients or healthy controls, between patients with and without inhaled corticosteroid usage, and between the patients that were frequent and non-frequent exacerbators (frequent exacerbators defined as exacerbation events > = 2/last year, Supplementary Figures S2A,B).

Of the 2,868 ASVs, 795 ASVs constituting 52.1% of all CCS reads were putatively assigned to 228 bacterial species in 92 genera (Supplementary Figure S3A). In comparison, using the MCSMRT pipeline, 499 out of 2,121 non-singleton OTUs constituting 44.8% reads were assigned to 164 bacterial species in 88 genera (Supplementary Table S3). Using the 99% OTU-based clustering approach, 681 out of 2,159 non-singleton OTUs constituting 39.0% reads were assigned to 110 bacterial species in 60 genera (Supplementary Table S3). The DADA2 approach outperformed the clustering-based approaches for our data in terms of the number of species and the proportion of reads assigned to species, and was chosen for downstream analyses. Twenty species had an average relative abundance greater than 0.005 (Table 2). The number of species capable of being detected increased by 3.26 folds compared to a re-analysis of all previous COPD airway microbiome studies using similar pipeline (Supplementary Table S4). *Streptococcus*, *Prevotella*, and *Neisseria* had the highest numbers of 26, 18 and 12 species identified (Supplementary Figure S3B). LEfSe analysis identified 11 discriminatory species-level taxa between COPD and controls (Figure 1C, LDA > 2.0). Random forest analysis using these 11 species yielded increased precision in classifying patients, compared to that using 9 genera obtained with the same criteria (LDA > 2.0) (Figure 1D and Supplementary Figure S3C, AUC: 0.787 versus 0.706, *P* = 0.026). Figure 1E showed an overview of species-level airway microbiome profile.

**TABLE 2:** The major species-level taxa identified in this study (average relative abundance > 0.005).

**Species**	**All average**	**COPD average**	**Healthy average**	**Fold change**	**FDR *P*-value**
*Prevotella intermedia*	0.005	0.006	0.005	1.191	0.319
*Prevotella melaninogenica*	0.060	0.057	0.071	0.801	0.342
*Prevotella pallens*	0.013	0.012	0.017	0.700	0.0140*
*Streptococcus pseudopneumoniae*	0.017	0.020	0.004	5.667	0.222
*Streptococcus salivarius*	0.013	0.015	0.004	3.664	0.277
*Streptococcus thermophilus*	0.027	0.030	0.014	2.228	0.809
*Haemophilus influenzae*	0.043	0.052	0.008	6.779	0.617
*Haemophilus parahaemolyticus*	0.009	0.010	0.004	2.564	0.204
*Haemophilus parainfluenzae*	0.012	0.009	0.020	0.474	0.0074**
*Neisseria meningitidis*	0.009	0.009	0.008	1.044	0.128
*Neisseria mucosa*	0.007	0.008	0.002	4.452	0.785
*Neisseria perflava*	0.011	0.012	0.005	2.391	0.742
*Neisseria subflava*	0.017	0.012	0.036	0.330	0.0415*
*Fusobacterium nucleatum*	0.016	0.013	0.027	0.495	0.139
*Fusobacterium periodonticum*	0.014	0.012	0.022	0.531	0.0199*
*Pseudomonas aeruginosa*	0.016	0.020	0.000	NA	0.105
*Moraxella catarrhalis*	0.030	0.038	0.001	28.947	0.275
*Veillonella parvula*	0.005	0.004	0.010	0.439	0.208
*Porphyromonas gingivalis*	0.010	0.009	0.015	0.570	0.282
*Campylobacter concisus*	0.006	0.006	0.007	0.902	0.233

*P*-value was calculated using Wilcoxon rank-sum test. ***FDR *P* < 0.001; **P < 0.01; *P < 0.05.

Among the species-level taxa, *Haemophilus parahaemolyticus* was significantly increased in COPD smokers versus COPD non-smokers (fold-change = 6.40, FDR *P* = 0.02, Supplementary Figure S2C). *Ralstonia mannitolilytica*, an opportunistic pathogen, was significantly increased in frequent exacerbators, a subgroup of COPD patients that experienced more frequent acute exacerbation events than others (fold-change = 4.94, FDR *P* = 0.005, Supplementary Figure S2C). The increase of *R. mannitolilytica* was further confirmed by qPCR (Supplementary Figure S2D).

There was an overall decreased alpha diversity (Shannon index) and non-significantly increased relative abundance of *Haemophilus* in patients with increased severity measured using both spirometry-based GOLD I-IV and new GOLD A-D classification scheme based on exacerbation frequency, CAT and mMRC scores [Global Initiative for Chronic Obstructive Lung Disease (GOLD), 2019], consistent with previous findings (Mayhew et al., 2018; Dicker et al., 2020) (Supplementary Figures S4A,B). No species-level taxa reached statistical significance in association with GOLD classification, CAT or mMRC scores (FDR *P* < 0.05). *Fusobacterium periodontium* exhibited significant positive correlation with pre-FEV_1_ (FDR *P* < 0.05, Supplementary Figure S4C).

In our study, 58 out of 98 patients were administered long-term ICS. We classified the 58 patients into subgroups based on their daily ICS doses (500 ug/day [*n* = 5], 320 ug/day [*n* = 15], 250 ug/day [*n* = 14], 160 ug/day [*n* = 17], and less than 160 ug/day [*n* = 7]). All five groups have comparable alpha diversity (Supplementary Figure S5A). No clear clustering was observed for these groups in principal coordinate analysis based on Bray-Curtis dissimilarity (Supplementary Figure S5B). No genus or species-level taxa were significantly different across these groups. There was a non-significant increase of *Moraxella* and *Pseudomonas* in the higher dose groups (500 ug and 320 ug groups, Supplementary Figure S5A), consistent with the notion that higher dose of ICS may increase pathogenic bacterial load (Contoli et al., 2017).

### Substantial Intra-Genus Variation in the Airway Microbiome

**FIGURE 2:**
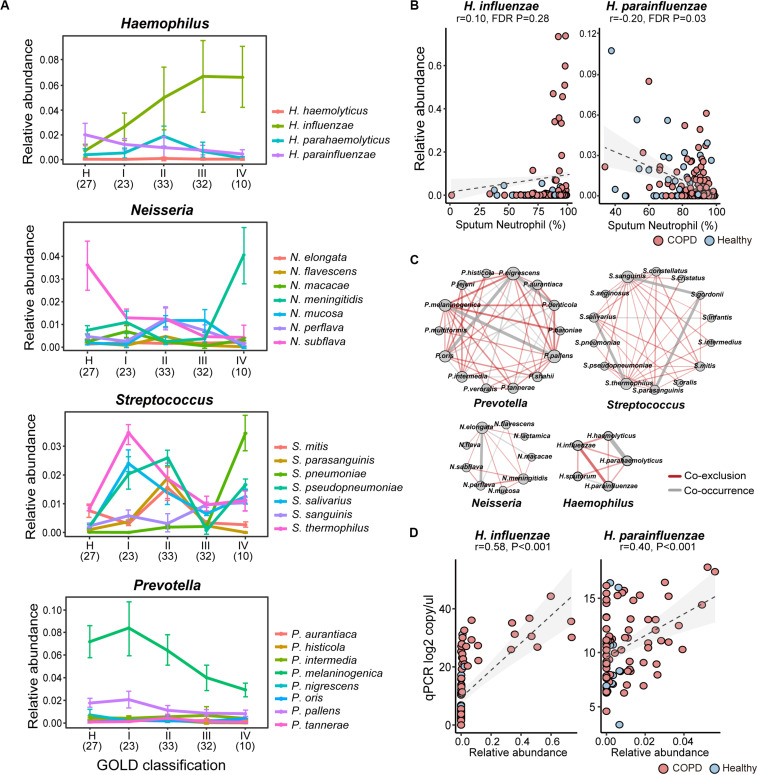
The intra-genus heterogeneity of the airway microbiome. **(A)** The alternation of major species in *Haemophilus*, *Neisseria*, *Streptococcus*, and *Prevotella* between healthy controls and COPD patients with increasing disease severity based on GOLD classification (spirometry-based). The average relative abundance and standard deviation of each species in each group were shown. The number of subjects in each subgroup was indicated in the parenthesis. **(B)** The reciprocal relationship between *H. influenzae* and *H. parainfluenzae* with sputum neutrophilic percentage. **(C)** The species-level co-occurrence network showed more pervasive co-exclusive than co-occurrence relationships between major species-level taxa within *Prevotella*, *Streptococcus*, *Neisseria* and *Haemophilus*. Each node represents a species-level taxon and each edge represents a correlation between paired taxa. Only significant correlations were shown in the networks (SparCC, *P* < 0.05). The size of the node is proportional to its degree of connectivity. The width of the edge is proportional to the absolute correlation coefficient. Co-exclusion relationships were colored in red, whereas co-occurrence relationships were colored in gray. **(D)** qPCR assays using species-specific primers showed concordance between absolute counts and relative abundances of *H. influenzae* and *H. parainfluenzae*.

Inspection of individual species revealed substantial intra-genus variation in their relationships with COPD. For example, while *Neisseria mucosa* was increased in COPD versus controls, its counterpart *Neisseria subflava* was significantly depleted (Figure 2A). The reciprocal relationships with COPD were also observed between *Haemophilus influenzae* and *Haemophilus parainfluenzae*, and between *Prevotella oris* and other *Prevotella* species (Supplementary Figure S6). The species also altered differentially with enhanced disease severity. For example, *H. parainfluenzae* and *N. subflava* were the most predominant species within the respective genera in healthy subjects, while *H. influenzae* and *N. meningitidis* were over-dominant in GOLD IV class of COPD patients with the highest disease severity (Figure 2A). Within *Streptococcus*, *Streptococcus salivarius* and *Streptococcus thermophilus* were most highly abundant in GOLD I patients, whereas *Streptococcus pseudopneumoniae* and *Streptococcus pneumoniae* were dominant in GOLD II and IV patients, respectively (Figure 2A). Opposite relationships were further observed between *H. influenzae* and *H. parainfluenzae* (Figure 2B), and between *Prevotella melaninogenica* and *Prevotella denticola* with patient sputum neutrophilic levels (Supplementary Figure S7). Individual species within the same genus exhibited disproportionately more co-exclusive than co-occurrence relationships (Figure 2C), indicating potential ecological interference. These results indicated that there was substantial intra-genus heterogeneity in the airways possibly resulting from interspecific competition. qPCR using primers designed on species-specific genes showed overall concordance between the absolute count and relative abundance of *H. influenzae* and *H. parainfluenzae* (Figure 2D), although the qPCR results showed better sensitivity at lower abundances.

### Major Airway Inflammatory Phenotypes Shaped Species-Level Microbiome-Host Interactions

**FIGURE 3:**
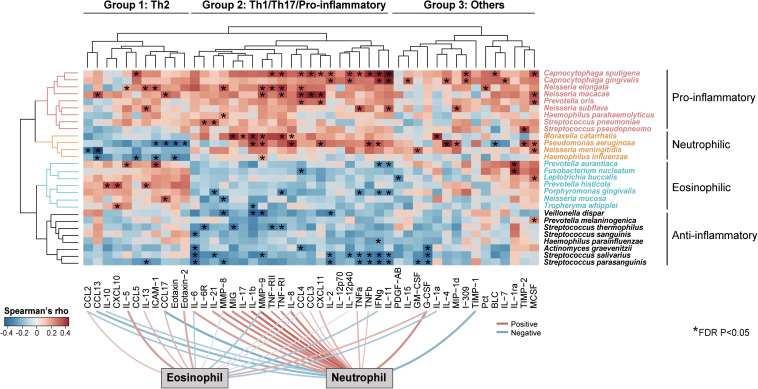
Species-specific association of airway microbiome with inflammatory phenotypes. Unsupervised two-way hierarchical clustering analysis on an all-against-all correlation profile between species-level microbiome features and 47 sputum mediators from a subset of 59 COPD patients (Ward’s method), in which microbiome features and mediators were both clustered. The species were shown if they had relative abundance > 0.001 and were significantly associated with at least one of the 47 sputum mediators (HAllA, FDR *P* < 0.05). The mediators were clustered into three groups and termed based on their classes and associations with airway eosinophils or neutrophils (Group 1: Th2-related, Group 2: Th1/Th17/Pro-inflammatory-related, Group 3: Others). The microbiome features were clustered into four groups based on their association patterns with the three groups of mediators (termed “Pro-inflammatory,” “Neutrophilic,” “Eosinophilic,” and “Anti-inflammatory”). The significant associations were indicated in asterisks. Significant positive and negative associations between sputum mediators and neutrophilic and eosinophilic percentages were shown on bottom of the heatmap (FDR *P* < 0.05).

To investigate how the intra-genus heterogeneity was related to airway inflammation, we performed an all-against-all correlation analysis between the species-level microbiome features and a panel of 47 sputum inflammatory mediators measured in a subset of 59 COPD patients. We used residualized correlation to identify microbiome-mediator correlations independent of patient demographic co-factors (Lloyd-Price et al., 2019). Unsupervised two-way hierarchical clustering based on the microbiome-mediator correlation profile revealed four clusters of bacterial species that each had distinct association patterns with three groups of mediators (Group 1–3, Figure 3). Four pathogens, *Moraxella catarrhalis, Pseudomonas aeruginosa, N. meningitidis* and *H. influenza*e, exhibited negative associations with a group of 11 mediators mostly Th2-related (i.e., IL-5, IL-13, CCL17), while they were positively correlated with a group of 21 mediators mostly Th1, Th17-related or pro-inflammatory (i.e., IL-8, IL-17, MMP-8), and had mixed relationships with the remaining mediators. By contrast, another seven species, *Prevotella aurantiaca, Fusobacterium nucleatum, Leptotrichia buccalis, Prevotella histicola, Porphyromonas gingivalis, N. mucosa* and *Tropheryma whipplei*, were specifically associated with increased Th2 mediators and decreased Th1/Th17/pro-inflammatory mediators. Members of these two groups of mediators further showed specific correlations with increased sputum neutrophils or eosinophils, respectively (FDR *P* < 0.05), in agreement with their roles in neutrophilic or eosinophilic inflammation, the two major airway inflammatory phenotypes in COPD (Balzano et al., 1999; Saha and Brightling, 2006). Correspondingly, all seven species were increased in the subgroup of COPD patients with high eosinophilic levels (sputum eosinophil > = 3%) versus those with low eosinophilic levels (sputum eosinophil < 3%, Supplementary Figure S8A). The increase of *T. whipplei* was further confirmed by qPCR (Supplementary Figure S8B). The other two groups of species in general showed consistent positive or negative associations with the broad panel of inflammatory mediators (denoted as ‘Pro-inflammatory’ or ‘Anti-inflammatory’ in Figure 3). Such phenotype-specific clustering pattern was not observed at the genus-level (Supplementary Figure S9), indicating that airway microbiome interacted with host in a species-specific manner, which was shaped by major airway inflammatory phenotypes.

### An Automated Pipeline for Strain-Level Identification in the Airway Microbiome

**FIGURE 4:**
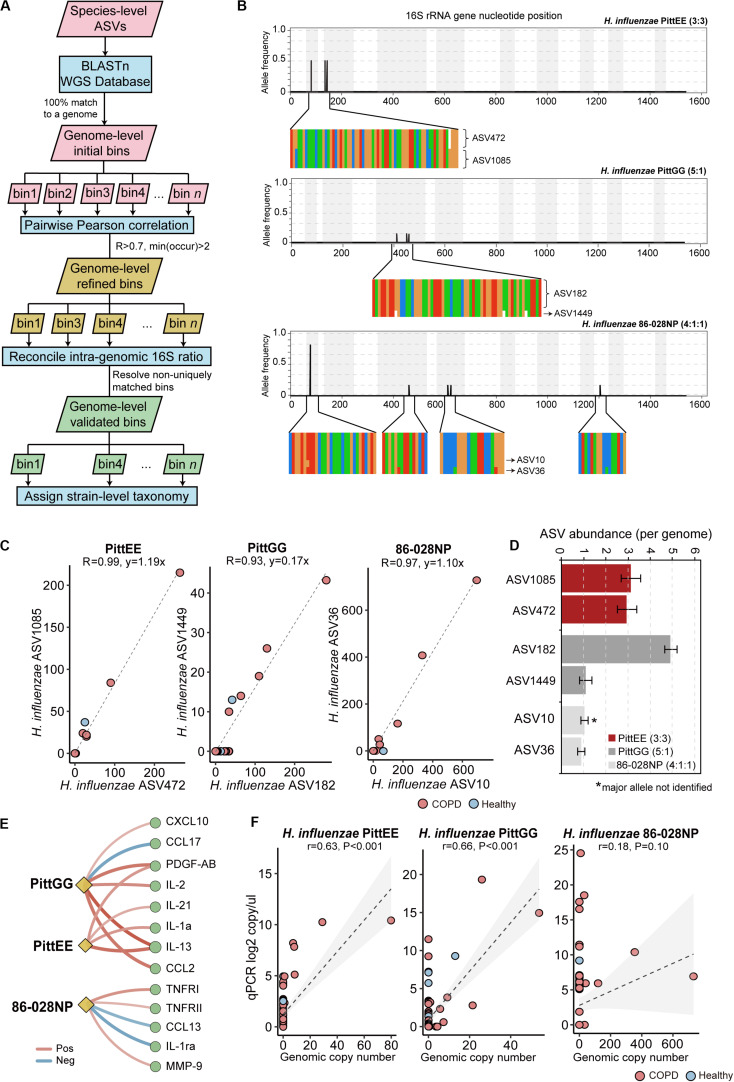
Strain-level identification in the airway microbiome. **(A)** The designed pipeline for identifying strain-level ASV bins. **(B)** The polymorphism in the 16S rRNA gene sequences for *H. influenzae* PittEE, PittGG and 86-028NP strains. The position and frequency of substitution in the full-length 16S sequences of the three strains were shown. The magnified regions showed respective positions in the alignment of all six copies of 16S gene in the corresponding *H. influenzae* genomes. The genuine ratios of 16S allelic variants in the three genomes are: 3:3, 5:1 and 4:1:1. **(C)** The correlation pattern between the counts for pairs of ASVs assigned to the strains PittEE, PittGG and 86-028NP (Pearson’s *R* > 0.93). **(D)** The copy number of the highly-correlated ASVs are in integral ratio with the genuine allelic frequency of the 16S rRNA genes within the genome. The major 16S allele of the 86-028NP strain was not detected. **(E)** Significant associations between the three *H. influenzae* strains with sputum mediators (Spearman, FDR *P* < 0.05). **(F)** qPCR results using strain-specific primers for the three *H. influenzae* strains in relation to their relative abundances in the sequencing data.

We further explored possible strain-level diversity in the airway microbiome. Recent studies showed that it is possible to resolve strain-level identity using full-length 16S sequences by leveraging the power of the full complement of 16S rRNA alleles within bacterial genomes (Callahan et al., 2019; Johnson et al., 2019). Callahan et al. introduced a simple rule to manually assign strain-level taxonomy by examining ASVs according to 16S integral ratio, similar taxonomy assignments and consistent patterns across samples (Callahan et al., 2019). In extension to this rule, we designed a pipeline to automate this process and identify strain-level ASV bins in a large-scale. Using this pipeline with a set of stringent criteria (see methods, Figure 4A), we identified genome-level ASV bins corresponding to 14 different bacterial strains with reasonable confidence (Supplementary Table S5). This included ASV bins with sequence identical and in integral copy number ratio to the 16S rRNA gene alleles of three non-typeable *H. influenzae* (NTHi) strains PittEE, PittGG and 86-028NP, although the major 16S allele of 86-028NP was not detected (Figures 4B–D). All three strains increased in COPD versus controls. The three strains were associated with distinct groups of mediators, implicating that they likely provoked different types of host inflammatory response (Figure 4E). In particular, 86-028NP and PittGG exhibited inverse associations with Th2 chemokines such as CCL17 and CCL13 related to eosinophilic inflammation. qPCR using strain-specific primers showed moderate concordance for PittEE and PittGG (Figure 4F), although the strain detection rate by sequencing was lower than that using qPCR. qPCR for 86-028NP yield positive but non-significant correlation (Figure 4F).

### A Systematic Evaluation of 16S Sub-Regions for Airway Microbiome Profiling

**FIGURE 5:**
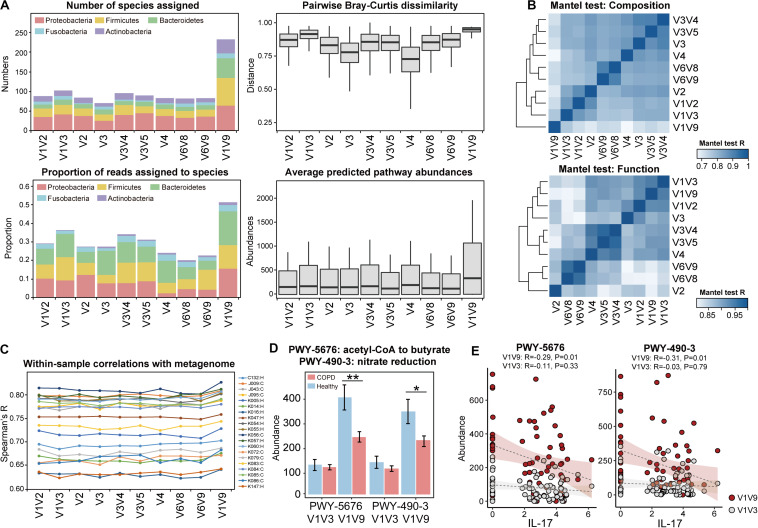
Systematic comparison of full-length 16S data and sub-region data for taxonomic diversity and functional inference. **(A)** Comparisons between the full-length 16S data and the sub-region data, in terms of (1) the number of species-level taxa assigned, (2) the proportion of reads that can be assigned to species, (3) the pairwise Bray-Curtis dissimilarity across all samples, and (4) the average abundances of pathways predicted by PICRUSt2. **(B)** Heatmap showing similarity in compositional and functional profiles between the full-length 16S data and data for individual 16S sub-regions. Mantel test for Bray-Curtis dissimilarity was performed to assess the profile-level correlations. **(C)** The correlation between PICRUSt2-inferred genes and the same genes in the actual metagenome, within each of the 20 samples. The sample IDs and their corresponding groups (COPD: **C**, Healthy: **H**) were indicated. **(D)** The PICRUSt2-inferred abundances of the two pathways ‘PWY-5676: acetyl-CoA fermentation to butyrate’ and ‘PWY-490-3: nitrate reduction’ in COPD and controls using the full-length (V1V9) and V1V3 data (** FDR *P* < 0.01, * FDR *P* < 0.05). **(E)** The two pathways showed negative correlations with IL-17, which was more pronounced when inferred from full-length 16S sequences than V1V3 sequences.

The full-length 16S sequences can serve as a benchmark for a systematic evaluation on the performance of individual hypervariable regions for airway microbiome studies. To this end, we created partitions of 16S sequences from the full-length data according to nine hypervariable regions used in previous COPD microbiome studies, and analyzed each partition separately. Among all sub-regions, V1V3 and V3V4 were among the highest in terms of the number of species assigned as well as the proportion of sequences assigned to species (Figure 5A). In addition, the V1V3 and V3V4 regions captured the greatest microbial beta diversity measured using pairwise Bray-Curtis dissimilarity, whereas the diversity was the lowest for the V4 region (Figure 5A). The V4 region was particularly poor in classifying Proteobacteria and Actinobacteria, with 79.8 and 90.9% of sequences from these two phyla unable to be assigned to species (Supplementary Figure S10). Mantel test for Bray-Curtis dissimilarity showed that the V1V3 region also bear the highest similarity with the full-length 16S data in the overall community composition (Figure 5B).

### Full-Length 16S Sequences Augmented the Power of Functional Inference

PICRUSt is a useful tool to infer functional capacity of microbiome based on 16S sequences (Douglas et al., 2020). We compared PICRUSt2 prediction results using the full-length 16S sequences and sequences from the nine hypervariable regions. PICRUSt analysis using the full-length 16S data greatly enhanced the power of functional inference by increasing the copy number abundances of the predicted pathways by an average 1.83-fold compared to individual sub-regions. Again, V1V3 were the next best in terms of the predicted pathway abundances (Figure 5A). The same sub-region also showed the highest similarity with the full-length 16S data in the inferred functional profile (Mantel test, Figure 5B).

To further assess the precision of functional inference using the full-length 16S data, we characterized the airway metagenome for a randomly selected set of 10 COPD patients and 10 controls as benchmark. We performed correlation analysis between genes in the actual metagenome and PICRUSt2-inferred metagenome using the full-length 16S data as well as sub-region data. When examining each sample, there was an overall correlation pattern between the PICRUSt2-inferred genes and those from the metagenomic data (Figure 5C, Supplementary Figure S11, Supplementary Table S5, Spearman’s R: 0.624–0.818). The correlations were notably centric to the abundant genes, whereas genes with less abundance showed more random patterns (Supplementary Figure S11). This suggested that PICRUSt2 better predicted the more abundant genes in the metagenome. PICRUSt2 inference based on full-length 16S sequences yielded slightly better per-sample correlations with metagenome than those based on sub-region sequences (i.e., Spearman’s R: 0.750 ± 0.06 for full-length data versus 0.736 ± 0.06 for V1V3 data, Figure 5C, Supplementary Table S6), as well as a higher resemblance to the sample-wise metagenome profiles based on Mantel test for Bray-Curtis dissimilarity (Supplementary Table S6). When examining each individual gene, PICRUSt2 inference using full-length 16S data generated more genes that showed correlations with metagenome across all samples, compared to the inferences using sub-region data (305 KO genes versus 248.2 ± 18.8 genes, Spearman’s *R* > 0.5, Supplementary Table S6).

At the pathway level, PICRUSt2 inference based on full-length 16S sequences led to slightly different set of pathways differentially abundant in COPD versus controls (FDR *P* < 0.05, Supplementary Table S7). This included the identification of 9 pathways that were uniquely associated with COPD when inferred using the full-length 16S data (Supplementary Table S8). Of interest are two pathways ‘acetyl-CoA fermentation to butyrate’ and ‘nitrate reduction,’ both inferred as significantly depleted in COPD (FDR *P* < 0.05, Figure 5D, Supplementary Figure S12). Both pathways were also decreased in COPD versus controls in the subset metagenomic data (log2 fold-change = −1.608 and −2.502 respectively, Supplementary Table S8). qPCR using validated broad-spectrum primers on butyryl-CoA:acetate-CoA-transferase gene (Vital et al., 2013) in the butyrate pathway further supported the finding by showing a 4.32-fold decrease of the gene in COPD versus controls (Supplementary Table S9). Furthermore, the two pathways showed inverse correlations with sputum inflammatory marker IL-17, which were more pronounced when inferred from full-length 16S data than from sub-regions (Figure 5E).

## Discussion

Here, we provided the first insights on the COPD airway microbiome at the species and strain-levels. By applying the ‘third-generation’ PacBio sequencing to the full-length 16S rRNA gene, we uncovered diversity and complexity in the airway microbiome at in-depth taxonomic levels that were previously underappreciated. In light of our results, many aspects of our understanding of the airway microbiome need to be refined.

Our results showed that there were substantial intra-genus variations in the airway microbiome in relation to patient clinical outcomes. Individual species within the same genus often altered differentially in COPD and with enhanced clinical severity. The species predominant in healthy state can be taken over by another species within the same genus but with potentially increasing pathogenicity in severe COPD patients. Understanding such variation is a prerequisite in precisely identifying and targeting key microbial species at play at different clinical stages of COPD. All previous studies by reporting the aggregated genus-level associations failed to capture such phenomenon. Thus those results by neglecting the species-level variations only represent an attenuated signal by the mixed effects of individual species within, and should be interpreted with caution.

Unsupervised clustering analysis demonstrated clear species-level microbiome-host interaction patterns according to neutrophilic or eosinophilic inflammation, the two major inflammatory phenotypes in COPD (Balzano et al., 1999; Saha and Brightling, 2006). The neutrophil-specific bacterial species included known respiratory pathogens including *H. influenzae*, *M. catarrhalis*, *N. meningitidis*, and *P. aeruginosa* in Proteobacteria that provoked pro-inflammatory host response (Sethi and Murphy, 2001). There is increasing evidence showing that airway microbiome is associated with inflammatory phenotypes of COPD patients. Several studies have showed that airway microbiome differs between exacerbation phenotypes in particular between bacterial-associated and eosinophilic exacerbations, with reduced microbial diversity and increased Proteobacteria in the former group (Wang et al., 2016, 2018; Mayhew et al., 2018). In this regard, our results are consistent and extend these findings by showing that specific Proteobacteria species may be key members contributing to enhanced neutrophilic over eosinophilic inflammations. The respiratory pathogens can frequently colonize and be persistent in the airways despite innate host immunity through various evasive pathogenic mechanisms. For example, non-typeable *H. influenzae* evades host immune recognition and clearance by neutrophil extracellular traps (NETs) through invading host epithelial cells, forming biofilms, altering gene expression and displaying surface antigenic variation (Ahearn et al., 2017). *P. aeruginosa* is able to modify its motility, alginate production, biofilm formation or susceptibility to host anti-microbial defenses to establish niches of persistent infection (Skopelja-Gardner et al., 2019). *N. meningitidis* can be resistant against NETs through a series of mechanisms including modification of LPS and escape from NET-mediated nutritional immunity (Lappann et al., 2013). A recent study showed that *M. catarrhalis* achieved its persistent colonization in COPD patients through regulating major adhesin Hag/MID (Murphy et al., 2019). Our data showed significant positive correlations between *M. catarrhalis* and *P. aeruginosa* with NET-triggering inflammatory mediators such as CXCL8, IL-1b and TNFa, supporting possible involvement of NETs in COPD host-microbiome interactions (Dicker et al., 2018). Further studies are warranted to investigate association between the species or strain-level airway microbiome with NETopathic inflammation in COPD (Uddin et al., 2019).

The eosinophil-specific species included *T. whipplei*, a clinically important species reported to be implicated in pneumonia (Bousbia et al., 2010), HIV infection (Lozupone et al., 2013) and eosinophilic, corticosteroid-resistant asthma (Goleva et al., 2013; Simpson et al., 2016). Thus the association between *T. whipplei* and eosinophilic inflammation may be a common signature across airway diseases. Such phenotype-specific host-microbiome interaction pattern was not detected at the broader genus-level, which supports the notion that phylogenetically diversified airway bacterial species may act as ecological “guilds” in response to specific types of environmental stimuli (here being the different types of host inflammations). The species delineation enabled an ecologically coherent view of airway microbiome according to host inflammatory phenotypes.

The full-length 16S sequencing is capable of resolving subtle nucleotide substitutions that exist between 16S gene copies within a bacterial genome. It has been shown that such intra-genomic variations, when properly accounted for, can aid in strain-level identification (Johnson et al., 2019). Callahan et al. introduced an approach by manually obtaining ASV copy number ratios and reconciling with the genuine 16S ratio of their corresponding bacterial genomes (Callahan et al., 2019). By curating the intra-genomic 16S variations for all complete bacterial genomes, we show that this process can be made fully automated and applied in real, complex microbial communities. We detected three NTHi clinical strains PittEE, PittGG and 86-028NP in the airway microbiome with reasonable confidence, and suggested that even within the pathogenic species there may be variations between strains in their relationships with airway inflammations (Chin et al., 2005). All three strains were initially isolated from otitis media patients (Harrison et al., 2005; Hogg et al., 2007; Arce et al., 2009). It has been shown that the PittGG strain, by possessing an extra cluster of 339 genes and a Hif-type pili structure, conveyed greater virulence than PittEE (Arce et al., 2009). qPCR assays based on *alpA* gene on this extra locus confirmed our results in PittGG quantification. While all three strains were related to increased Th1/Th17 mediators, 86-028NP and PittGG were further associated with decreased Th2-related CCL13 and CCL17, indicating their abundances may inversely predict eosinophilic inflammation. The microbiome species or strains may be suitable markers for airway inflammatory phenotypes.

We identified *Ralstonia mannitolilytica* as significantly increased in a subgroup of COPD patients that experienced more frequent acute exacerbations than others, a clinically important phenotype in COPD for which the underlying pathophysiology was not completely understood (Wedzicha et al., 2013). *R. mannitolilytica* is an opportunistic pathogen that has been recovered from cystic fibrosis airways (Coenye et al., 2002). In a previous report, the same species was isolated from one COPD exacerbation patient in western China with extreme symptoms and acute respiratory failure (Zong and Peng, 2011). *Ralstonia* spp. rarely cause infection in healthy individuals but can be a severe pathogen especially in immunosuppressed patients (Basso et al., 2019). Therefore, the presence of *R. mannitolilytica* in stable COPD patients may be a risk factor in predisposing patients to recurrent infection and exacerbations.

There is emerging evidence showing that airway microbiome is associated with clinical outcome and mortality of COPD patients. Leitao Filho et al. found that microbiome dysbiosis, in particular the presence of *Staphylococcus* and absence of *Veillonella*, was associated with increased 1-year mortality for hospitalized AECOPD patients (Leitao Filho et al., 2018). *Staphylococcus* spp. were barely detected in our patients (3 out of 98 COPD patients) so their association with clinical outcomes were unable to be assessed in our cohort. A key difference between our study and Leitao Filho et al. is that only patients with severe exacerbations that required hospitalization were included in their study, whereas we captured mild, moderate and severe patients at clinical stability. Nevertheless, both our results (increased *Ralstonia mannitolilytica* in frequent exacerbators) and those of Leitao Filho et al. supported the notion that identification of rare opportunistic pathogens may be associated with worse clinical outcome and prognosis of COPD patients. A recent study by Dicker et al. showed that *Haemophilus* dominance at COPD clinical stability was linked to increased severity, frequent exacerbations and increased mortality (Dicker et al., 2020). This is also consistent with our finding that increased relative abundance of *Haemophilus*, in particular *H. influenzae* species, was observed in patients with enhanced GOLD status.

The systematic comparison of individual 16S sub-regions indicated that V1V3 region performed the best in terms of the species-level microbial alpha and beta diversity, the power of functional inference, as well as the profile resemblance with full-length 16S data in terms of both microbiome composition and function. Our results based on real sequencing data on natural microbial communities are consistent with the *in silico* analysis by Johnson et al. based on existing 16S databases (Johnson et al., 2019), and should guide future studies that sequencing V1V3 may be a surrogate for airway microbiome when sequencing the full-length 16S gene is not available. Conversely, sequencing the V4 region alone, despite its wide usage in previous airway microbiome studies, might not provide sufficient resolution for in-depth taxonomic profiling in particular in Proteobacteria and Actinobacteria.

The full-length 16S sequences yielded increased abundances for genes and pathways capable of being predicted, possibly due to the higher precision of full-length 16S sequences in hidden state prediction in the PICRUSt2 algorithm[24]. The augmented power led to better concordance with the actual metagenomes in a subset of 20 samples, thereby also increased the accuracy of functional inference. With better power, we inferred butyrate-producing and nitrate reduction pathways as uniquely depleted in COPD using the full-length 16S data. The decreases of these pathways were supported by the subset of samples with metagenomic data as well as some preliminary qPCR results. Butyrate is a well-characterized microbial metabolite with anti-inflammatory effects in human gut (Cait et al., 2018), whereas its role in the airways remains unclear. Nitric oxide, the end product of nitrate reduction, may have possible disease-ameliorating effects by suppressing NLRP3 inflammasome activation in the airways (Mao et al., 2013; Lee et al., 2016). The mechanistic roles of these pathways in COPD remain to be determined.

There are several caveats to our study. First, the study design is single-centered and cross-sectional so the temporal stability of the airway microbiome is unable to be tracked. Nevertheless, the major microbiome composition in our cohort, as well as the associations between key microbiome taxa and neutrophilic and eosinophilic inflammations, were generally consistent with previous findings on European and US cohorts, suggesting that there may be a common core airway flora and microbiome-host interactions despite geographical differences. Additional multi-centered cohorts preferably across continents (i.e., similar as the design in Mac Aogain et al., 2018) with longitudinal follow-up are needed to test this hypothesis and further validate our findings. Second, despite multiple approaches and databases employed, only about half of all CCS reads in our data can be confidently assigned to bacterial species-level taxa. This was also reflected by the lower sensitivity for species and strains detection based on sequencing compared to that based on qPCR. This is an important caveat due to inherently limited power of 16S sequences for species or strain resolution, its sensitivity to potential sequencing errors and incompleteness of current reference databases. Therefore even the full-length 16S gene survey may still underrepresent the true microbial species diversity in the airways. Third, despite efforts made in quality control and careful selection of sputum plugs from saliva, potential sources of oral contamination cannot be fully excluded. Comparative studies with concurrent oral rinse sampling are needed to distinguish bacterial species and strains originated from oral cavity and airways. Fourth, we lack sufficient data to explore species-specific relationships with other COPD etiological factors such as viral infections.

In summary, we present a finer-scale taxonomic analysis on the COPD airway microbiome. We showed there were substantial microbial diversity and heterogeneity at species-level and below, which was associated with patient clinical outcome and host inflammation. Sequencing the full-length 16S rRNA gene enabled a refined view on the composition and function of the airway microbiome in COPD, and should see a wider applicability in airway microbiome studies in future.

## Author’s Note

This manuscript has been released as a pre-print at BioRxiv (Wang et al., 2020).

## Data Availability Statement

The datasets generated for this study can be found in the China National GeneBank (CNGB) Nucleotide Sequence Archive (CNSA) CNP0000837.

## Ethics Statement

The studies involving human participants were reviewed and approved by the ethics committee of the First Affiliated Hospital of Guangzhou Medical University (No. 2017-22). The patients/participants provided their written informed consent to participate in this study.

## Author Contributions

ZW conceived and designed the study. HL, FW, YY, and ZL coordinated the collection of sputum samples and clinical data. HL, XW, and BC processed the sputum samples, performed the DNA extraction, and library preparation. ZW performed all data analysis. MS, CB, and WS provided key insights to the work. RC and HZ supervised the study. ZW wrote the manuscript. All authors provided critical comments and approved the final version of the manuscript.

## Conflict of Interest

The authors declare that the research was conducted in the absence of any commercial or financial relationships that could be construed as a potential conflict of interest.

## Footnotes

1 https://benjjneb.github.io/dada2/assign.html

2 https://github.com/wangzlab/Strain_ID_FL16S

3 http://primer3.ut.ee
