# Supplementary material for: A Refined View of Airway Microbiome in Chronic Obstructive Pulmonary Disease at Species and Strain-Levels
Source: Front Microbiol. 2020 Jul 30;11:1758. doi: 10.3389/fmicb.2020.01758 (PMC7406711; doi:10.3389/fmicb.2020.01758)
Supplement: Supplementary file 1 [file Data_Sheet_1.docx]

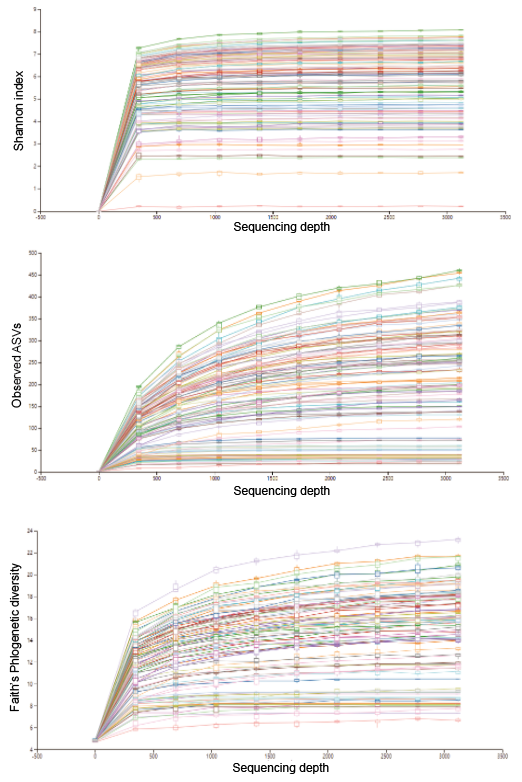


**Figure S1.** Alpha-rarefaction curve based on Shannon index, observed ASVs and Faith’s phylogenetic diversity. All samples were rarefied at the depth of 3,119 reads.


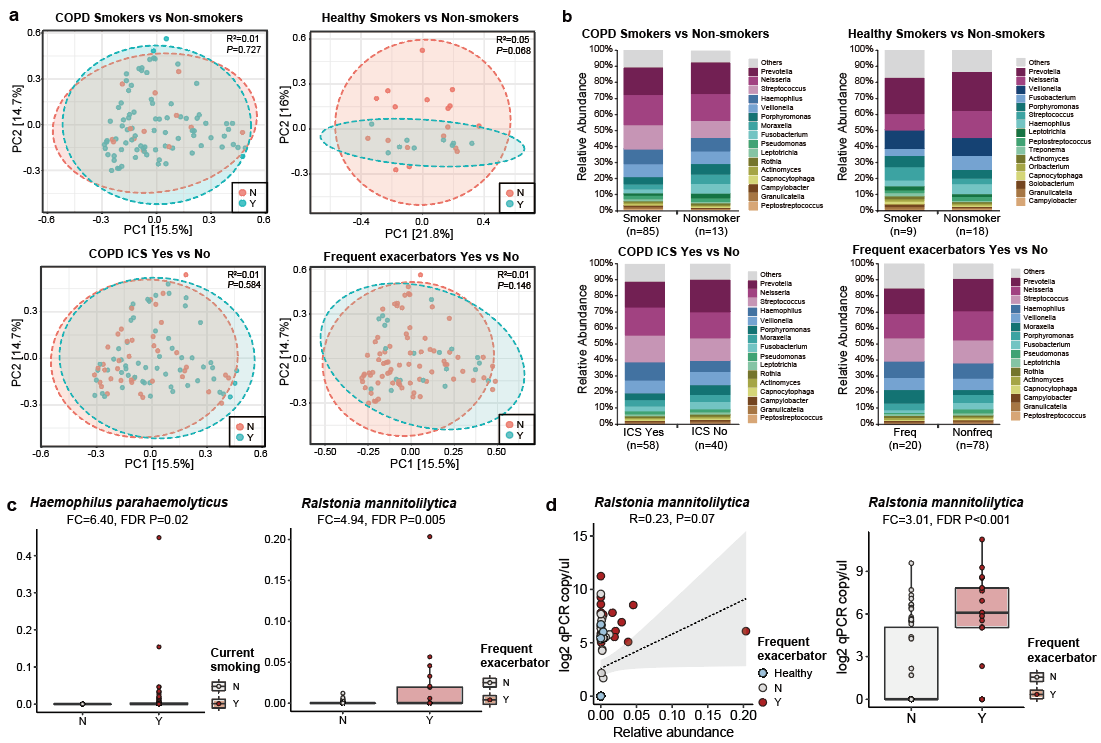


**Figure S2. a)** Principal coordinate analysis plots and **b)** genus-level microbiome profiles between COPD smokers versus non-smokers, healthy smokers versus non-smokers, COPD inhaled corticosteroid takers versus non-takers, and COPD frequent (defined as exacerbation events >=2/last year) and non-frequent exacerbators. **c)** Significant increase of *Haemophilus parahaemolyticus* in COPD smokers verus non-smokers (Fold-change=6.40, FDR *P*=0.02). Significant increase of *Ralstonia mannitolilytica* in frequent versus non-frequent exacerbators (Fold-change=4.94, FDR *P*=0.005, Figure S3c). **d)** qPCR assay based on species-specific primers for *Ralstonia mannitolilytica* confirmed the sequencing results.


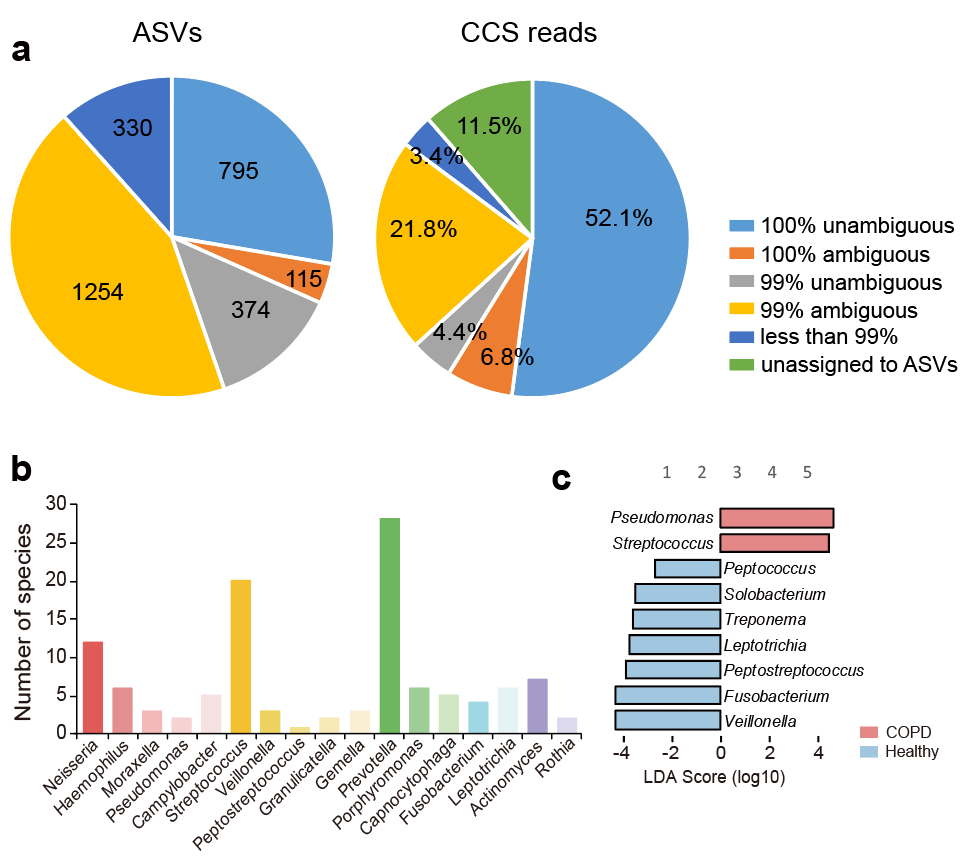


**Figure S3. a)** The distribution of ASVs and their corresponding CCS read counts in proportion to all quality-filtered CCS reads, according to their sequence matches in the NCBI nt database. **b)** The number of species identified for each genus in the full-length 16S rRNA gene sequencing data. **c)** The 9 discriminatory genus-level taxa between COPD and controls identified using LEfSe (LDA>2.0).


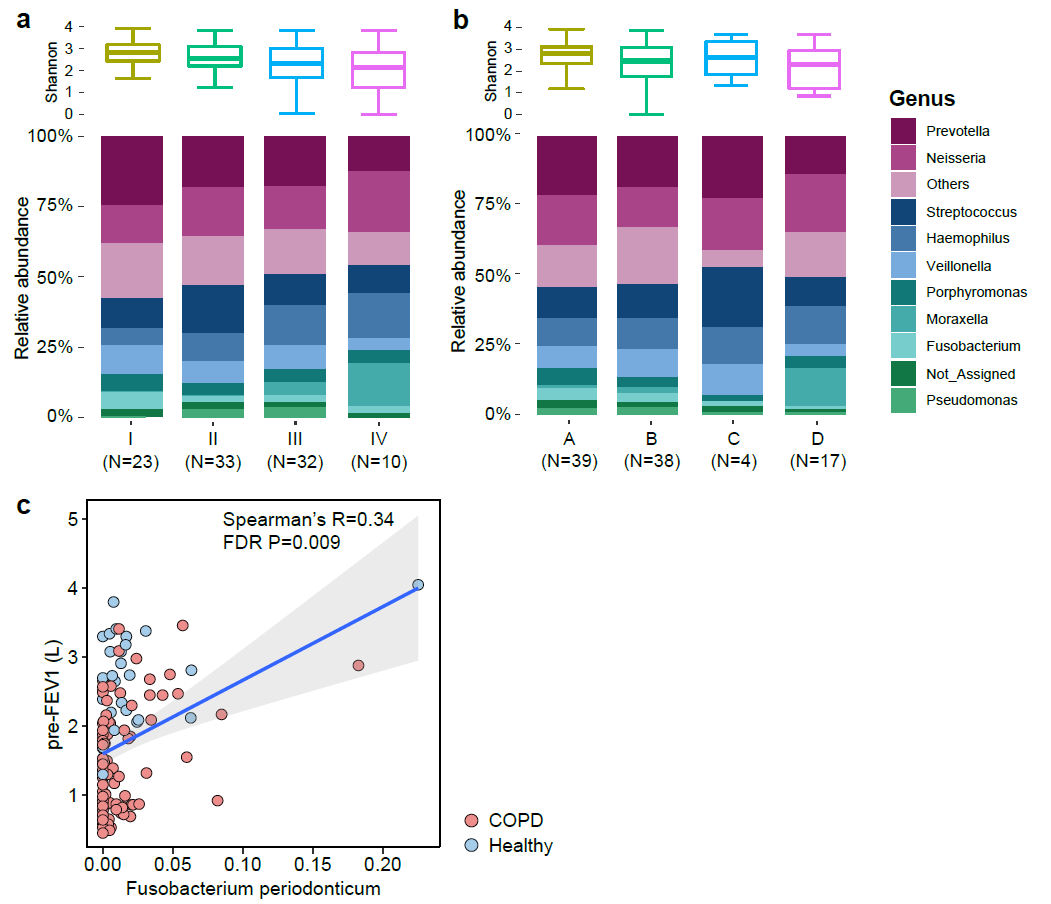


**Figure S4. a-b)** Alpha diversity (Shannon index) and main taxonomic distribution for patients with different levels of disease severity, classified using **a)** spirometry-based GOLD classification scheme, and **b)** new GOLD A-D classification scheme based on exacerbation frequency, CAT and mMRC scores. **c)** Significant positive correlations between *Fusobacterium periodontium* and pre-FEV1 (L).


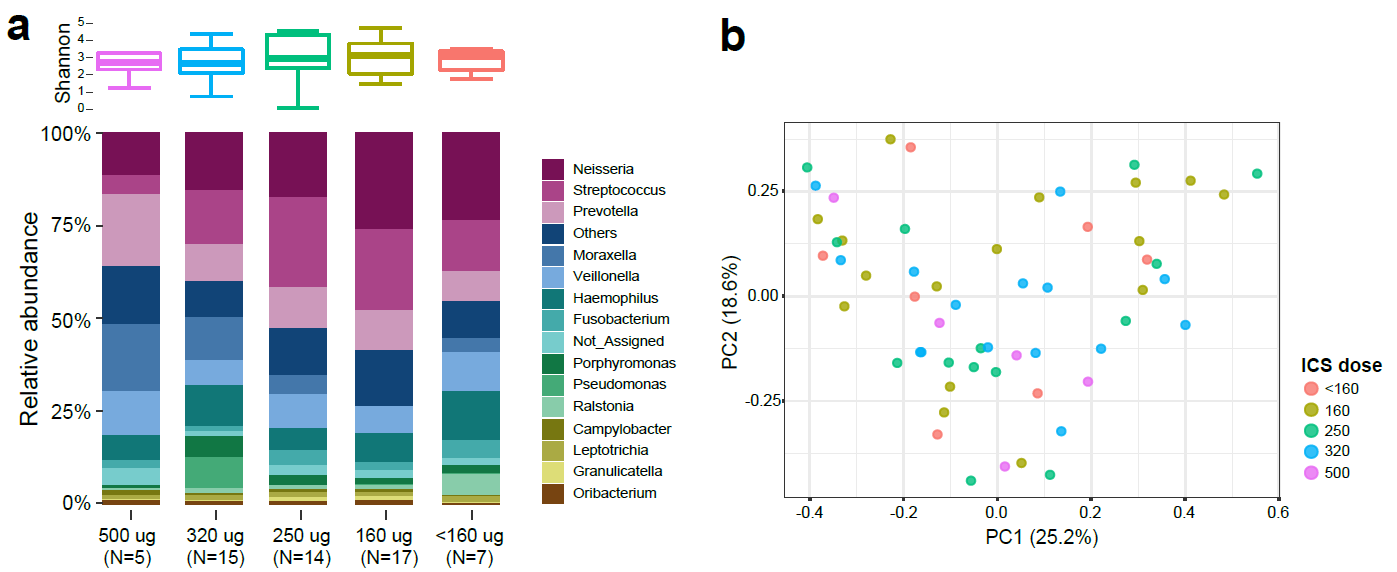


**Figure S5. a)** Alpha diversity (Shannon index) and main taxonomic distribution for 58 patients with different daily doses of ICS usage. **b)** Principal coordinate analysis of 58 patients based on Bray-Curtis dissimilarity. Patients were colored according to their corresponding ICS daily doses.


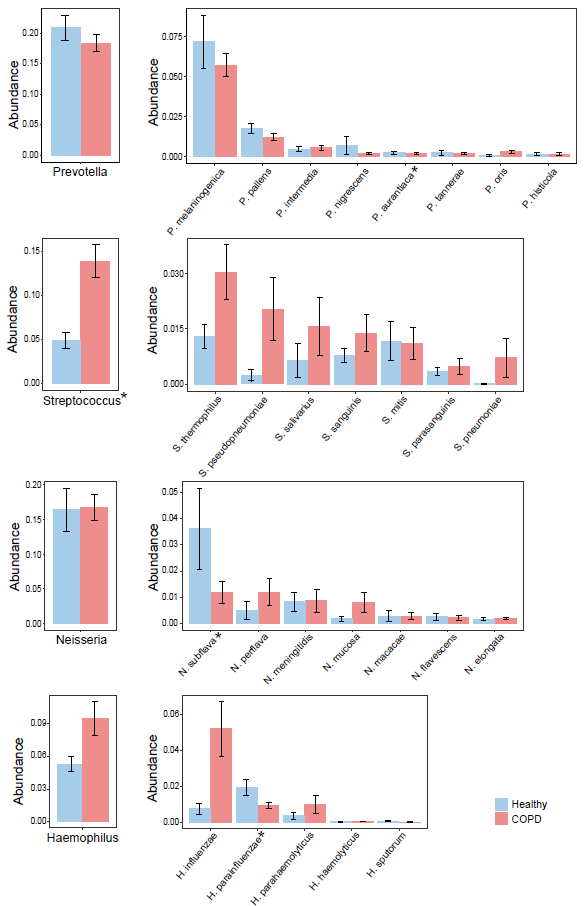


**Figure S6.** Heterogeneity in the changes in relative abundance of individual species (relative abundance>0.005) within *Prevotella*, *Streptococcus*, *Neisseria* and *Haemophilus* in COPD versus healthy controls. The average and standard deviation for each species in each group are shown. * FDR *P*<0.05


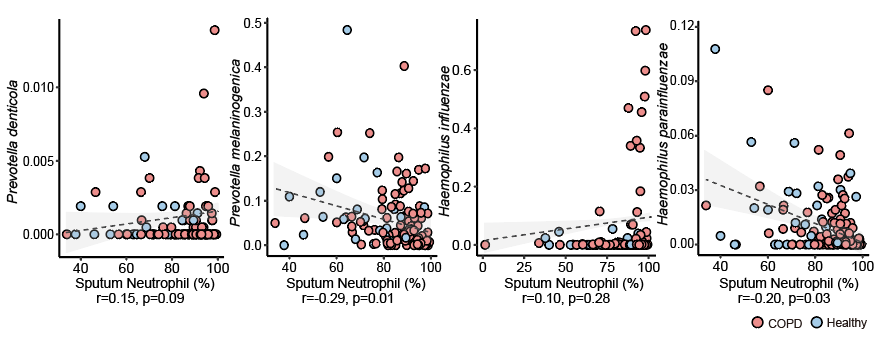


**Figure S7.** The reciprocal relationship between *Prevotella denticola* and *Prevotella melaninogenica*, and between *Haemophilus influenzae* and *Haemophilus parainfluenzae* and sputum neutrophilic percentage.


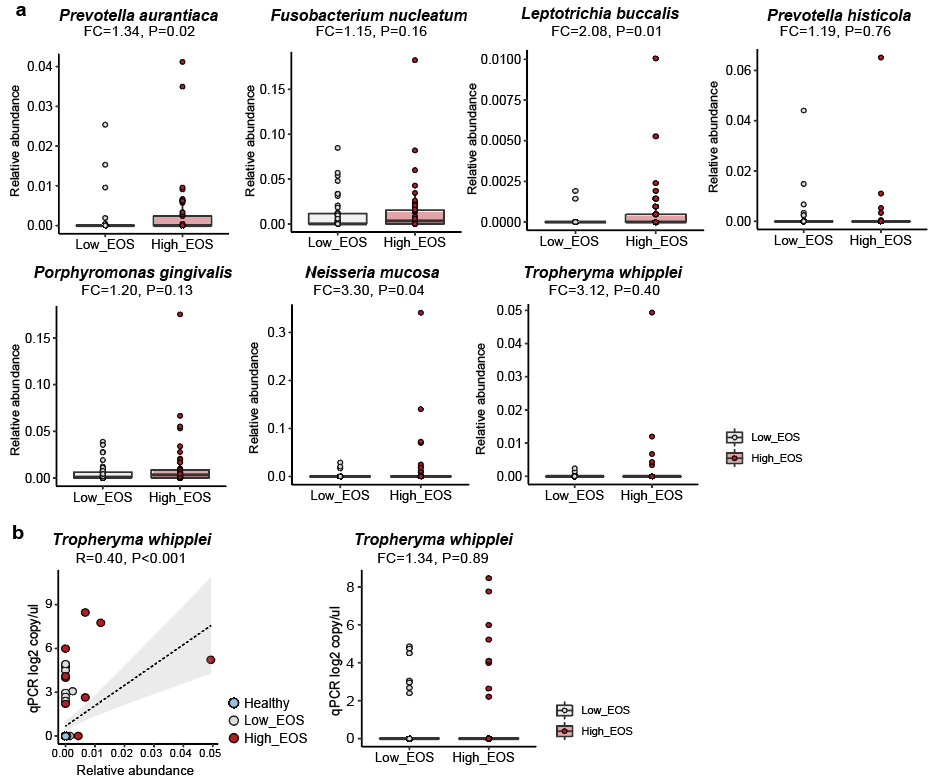


**Figure S8.** **a)** The alternations of the seven bacterial species associated with eosinophilic inflammations between patients with low eosinophilic levels (sputum EOS<3%) and high eosinophilic levels (sputum EOS>=3%). **b)** qPCR assays on *T. whipplei* confirmed the sequencing results.


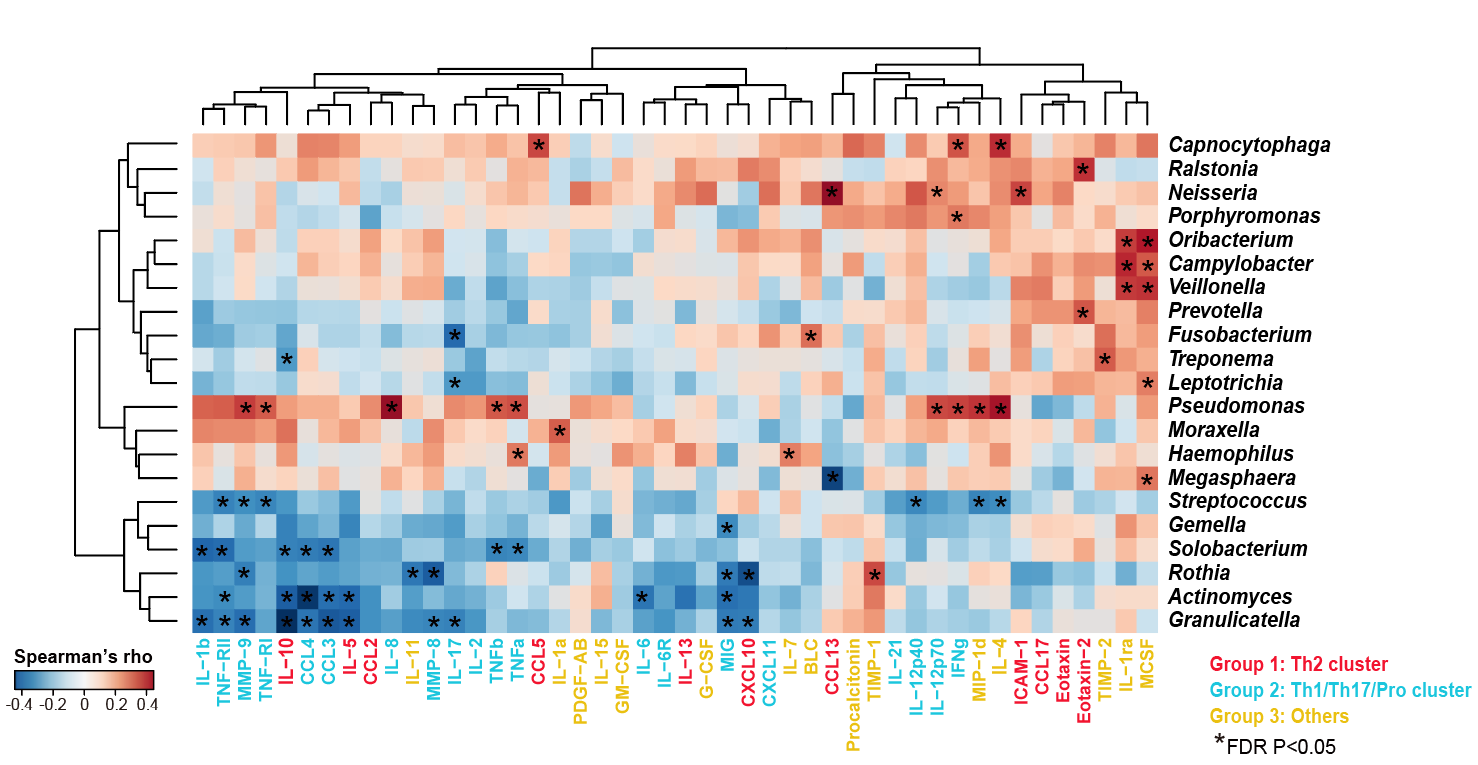


**Figure S9.** Clustered heatmap on correlations between genus-level microbiome profiles with the panel of 47 sputum mediators from a subset of 59 COPD patients. The genera were shown if they had relative abundance>0.001 and were significantly associated with at least one of the 47 sputum mediators (HAllA, FDR *P*<0.05). The significant correlations were indicated in asterisks. The 47 mediators were colored based on the assigned clusters (Group 1-3) from the correlation profile with the species-level microbiome features (Figure 3). No inflammatory phenotype-related clusters were observed for the genus-level associations.


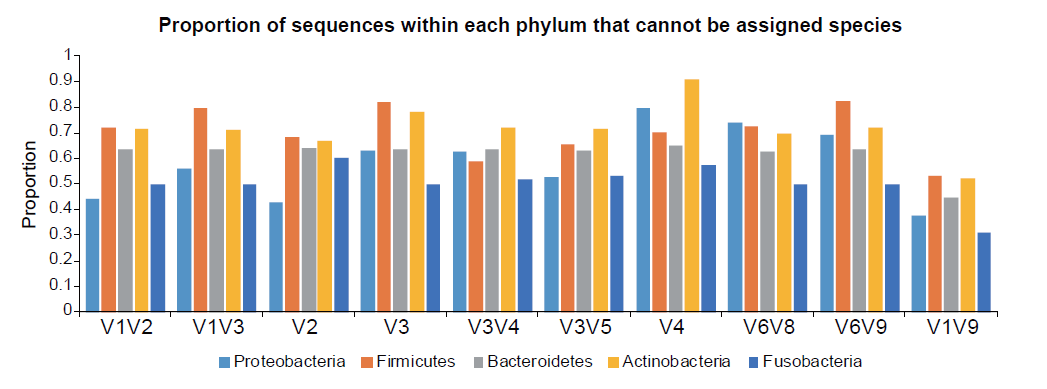


**Figure S10.** The proportion of sequences within each major phylum (Proteobacteria, Firmicutes, Bacteroidetes, Actinobacteria and Fusobacteria) that cannot be assigned with species-level taxa for the full-length sequences (V1V9) and nine individual hypervariable regions.


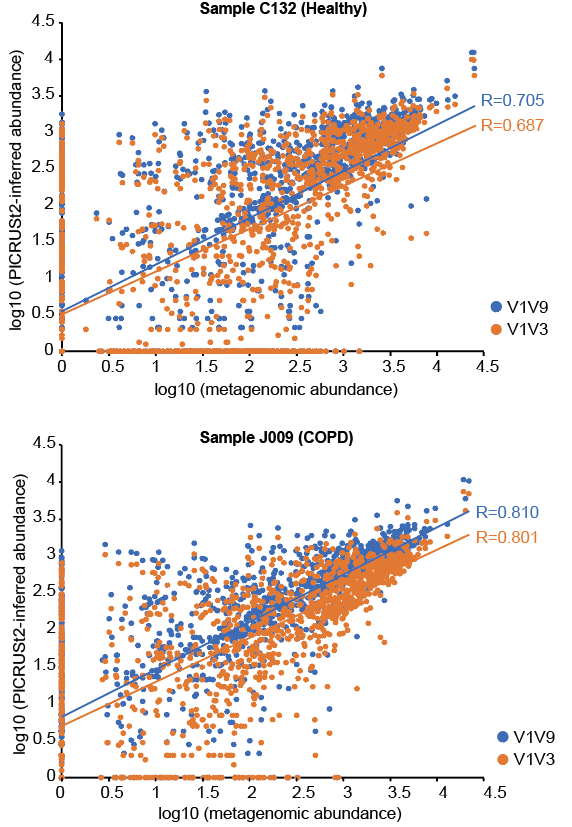


**Figure S11.** Correlation between PICRUSt2-inferred genes using full-length 16S data (V1V9) and V1V3 data and the same genes in the actual metagenomes. Correlation plots were shown for one healthy sample (C132) and one COPD sample (J009).


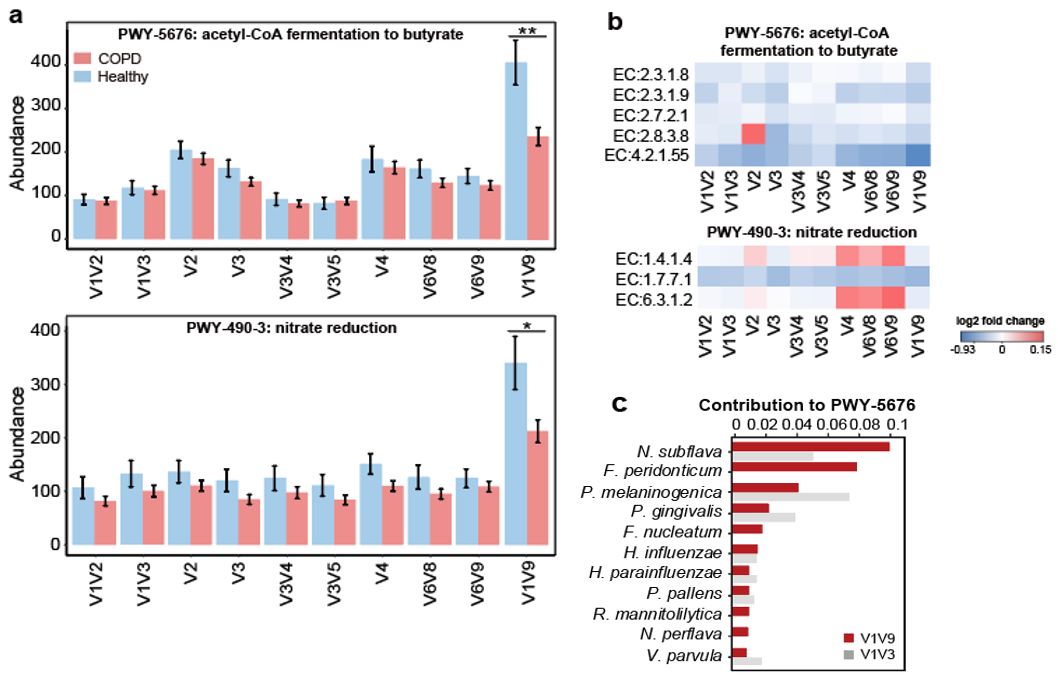


**Figure S12.** Functional inference using full-length 16S sequences and individual hypervariable regions using PICRUSt2. **a)** The inferred abundance in COPD and controls of pathways PWY-5676 (acetyl-CoA fermentation to butyrate) and PWY-490-3 (nitrate reduction) using the full-length 16S data (V1V9) and data for individual hypervariable regions. **b)** The inferred fold changes of individual genes within each pathway in COPD patients versus healthy controls. **c)** The top contributing bacterial species and their proportions of contribution to the ‘PWY-5676’ pathway as inferred from V1V9 and V1V3 data.

**Table S1.** The list of species-specific and strain-specific primers used in this study.

| **Species and strain** | **Target gene** | **Forward primer** | **Reverse primer** | **Size (bp)** |
| --- | --- | --- | --- | --- |
| *H. influenzae* | WP_005652235 outer membrane protein P6 | TTGGCGGWTACTCTGTTGCT | TGCAGGTTTTTCTTCACCGT | 296 |
| *H. parainfluenzae* | WP_014063976 hypothetical protein | TTCTACAGGCGGCCAAACGG | TCGGTTTCTCATCGGGTGGCA | 114 |
| *H. influenzae* PittEE | WP_005686347 hypothetical protein | TCAGTACTTTCGGCAACGTGGT | AGAGGCTACAGGAATCGGAGGA | 175 |
| *H. influenzae* PittGG | WP_012054916 AlpA family phage regulatory protein | AGCGAGGCAATTTTCCGAAGC | GCCATGCTGCGCCTCTTGTT | 119 |
| *H. influenzae* 86-028NP | WP_005672606 hypothetical protein | GCCTTACTGCCGTTTGTTTCGCA | GCACCGTCAGCTCCCTATGCA | 115 |
| *R. mannitolilytica* | AJW44311.1 hypothetical protein | ATGCCCCATTCCGTCAGCTT | AACACGCGCATCCCATGAAG | 154 |
| *T. whipplei* | WP_011102574 heat shock protein 65 | TGACGGGACCACAACATCTG | ACATCTTCAGCAATGATAAGAGAAGTT | 503 |

**Table S2.** All the ASVs and their numbers of sequences identified in the reagent controls.

| **ASV** | **DNA extraction blank** | **PCR blank** | **Taxonomy** |
| --- | --- | --- | --- |
| ASV1 | 32 | 0 | k__Bacteria; p__Proteobacteria; c__Alphaproteobacteria; o__Rhizobiales; f__Hyphomicrobiaceae; g__Devosia; s__ |
| ASV2 | 12 | 18 | k__Bacteria; p__Proteobacteria; c__Alphaproteobacteria; o__Rhodobacterales; f__Rhodobacteraceae; g__; s__ |
| ASV3 | 8 | 16 | k__Bacteria; p__Planctomycetes; c__Phycisphaerae; o__MSBL9; f__; g__; s__ |
| ASV4 | 5 | 9 | k__Bacteria; p__Proteobacteria; c__Gammaproteobacteria; o__Thiotrichales; f__Piscirickettsiaceae; g__; s__ |
| ASV5 | 2 | 1 | k__Bacteria; p__Proteobacteria; c__Deltaproteobacteria; o__Syntrophobacterales; f__Syntrophobacteraceae; g__; s__ |
| ASV6 | 1 | 0 | k__Bacteria; p__Chloroflexi; c__S085; o__; f__; g__; s__ |
| ASV7 | 0 | 2 | k__Bacteria; p__OP11; c__WCHB1-64; o__; f__; g__; s__ |

**Table S3.** The number of species-level, non-singleton ASVs/OTUs and their totaling proportion of reads generated using three different approaches, DADA2, MCSMRT and QIIME2 (99% OTU clustering using USEARCH).

| **Analytical approach** | | **DADA2** | **MCSMRT** | **QIIME2** |
| --- | --- | --- | --- | --- |
| Total number of ASVs/OTUs | | 2868 | 2121 | 2159 |
| Number of species-level ASVs/OTUs | All phyla | 795 | 499 | 681 |
|  | Firmicutes | 178 | 112 | 180 |
|  | Proteobacteria | 168 | 161 | 148 |
|  | Bacteroidetes | 255 | 113 | 279 |
|  | Actinobacteria | 60 | 21 | 10 |
|  | Fusobacteria | 95 | 24 | 0 |
| Number of species-level taxa | All phyla | 228 | 164 | 110 |
|  | Firmicutes | 72 | 42 | 29 |
|  | Proteobacteria | 67 | 42 | 27 |
|  | Bacteroidetes | 45 | 47 | 11 |
|  | Actinobacteria | 27 | 27 | 9 |
|  | Fusobacteria | 13 | 9 | 0 |
| Total proportion of reads assigned to species | | 52.1% | 44.8% | 39.0% |

**Table S4.** The number of species-level taxa identified using similar pipeline (the DADA2 pipeline customized for 454 or Illumina platforms) for all previous COPD airway microbiome datasets sequencing individual hypervariable regions of the 16S rRNA gene.

| **Accession** | **Sample type** | **PMID** | **Platform** | **16S region** | **Sample size** | **Number of species identified** |
| --- | --- | --- | --- | --- | --- | --- |
| SRP102480 | Sputum | 29269441 | Miseq | V4 | 446 | 104 |
| SRP102629 | Sputum | 29386298 | Miseq | V4 | 423 | 106 |
| SRP073159 | Sputum | 29101284 | Miseq | V3-V4 | 134 | 82 |
| SRP065072 | Sputum | 26917613 | 454 | V3-V5 | 106 | 116 |
| ERP108788 | Sputum | NA | Miseq | V3-V4 | 95 | 125 |
| Dyrad.5GC82 | Sputum | 28851370 | Miseq | V3-V4 | 81 | 120 |
| SRP066375 | Sputum | NA | Miseq | V4 | 181 | 112 |
| ERP022665 | BAL | 29992131 | 454 | V3-V5 | 64 | 82 |
| ERP003401 | Sputum | 25253795 | 454 | V1-V2 | 31 | 21 |
| ERP014054 | Sputum | NA | 454 | V6-V8 | 28 | 30 |
| SRP124904 | Sputum | 29518088 | 454 | V3-V4 | 26 | 18 |
| SRP064237 | Bronchial | 28704452 | 454 | V6-V9 | 39 | 41 |
| SRP065328 | BAL | 27486204 | Miseq | V4 | 20 | 13 |
| SRP057611 | Bronchial | 27146202 | Miseq | V1-V2 | 37 | 81 |
| SRP122946 | Bronchial | 29316977 | Miseq | V3 | 18 | 107 |
| SRP107187 | Sputum | 29579057 | 454 | V6-V8 | 14 | 54 |
| SRP075523 | Sputum | 27428540 | Miseq | V4 | 8 | 28 |
| SRP068430 | Bronchial | NA | Miseq | V2 | 12 | 28 |

**Table S5.** The list of strain-level ASV bins corresponding to 14 strain-level taxa identified using the pipeline designed in this study.

| **Species** | **Genome (Strain) best hit** | **ASVs in the bin** | **Pearson’s R** | **Genuine allele variants** |
| --- | --- | --- | --- | --- |
| *Capnocytophaga gingivalis* | H1496 | ASV5254 | NA | 1 |
| *Fusobacterium nucleatum* | subsp. vincentii | ASV1183;ASV835 | 0.97 | 1:1:1 |
| *Fusobacterium periodonticum* | KCOM 1261 | ASV141;ASV39 | 0.85 | 1:1:1 |
| *Haemophilus influenzae* | PittEE | ASV472;ASV1085 | 0.99 | 3:3 |
| *Haemophilus influenzae* | 86-028NP | ASV10;ASV36 | 0.97 | 4:1:1 |
| *Haemophilus influenzae* | PittGG | ASV182;ASV1449 | 0.93 | 5:1 |
| *Haemophilus parainfluenzae* | T3T1 | ASV272;ASV335 | 0.82 | 4:1:1 |
| *Neisseria cinerea* | NCTC10294 | ASV1047 | NA | 1 |
| *Neisseria elongata* | M15910 | ASV5395 | NA | 1 |
| *Neisseria sp.* | KEM232 | ASV496 | NA | 1 |
| *Prevotella jejuni* | CD3:33 | ASV180;ASV207 | 0.81 | 1:1:1:1 |
| *Streptococcus pseudopneumoniae* | IS7493 | ASV30 | NA | 1 |
| *Streptococcus salivarius* | NCTC7366 | ASV17;ASV53 | 0.75 | 5:1 |
| *Veillonella dispar* | NCTC11831 | ASV123;ASV191 | 0.72 | 1:1:1:1 |

**Table S6.** The Spearman correlation coefficients between metagenome genes and PICRUSt2-inferred genes based on full-length 16S data (V1V9) as well as sub-region data. Also included are the similarity of the overall functional profiles based on Mantel test for Bray-Curtis dissimilarity, and the number of PICRUSt2-inferred genes that showed correlations (Spearman’s R>0.5) with the metagenome across samples.

| **SampleID** | **Group** | **V1V2** | **V1V3** | **V2** | **V3** | **V3V4** | **V3V5** | **V4** | **V6V8** | **V6V9** | **V1V9** |
| --- | --- | --- | --- | --- | --- | --- | --- | --- | --- | --- | --- |
| C132 | Healthy | 0.697 | 0.687 | 0.694 | 0.692 | 0.694 | 0.693 | 0.698 | 0.699 | 0.700 | 0.705 |
| J009 | COPD | 0.801 | 0.801 | 0.800 | 0.798 | 0.803 | 0.805 | 0.802 | 0.799 | 0.801 | 0.812 |
| J043 | COPD | 0.778 | 0.770 | 0.778 | 0.777 | 0.786 | 0.783 | 0.773 | 0.786 | 0.782 | 0.793 |
| J095 | COPD | 0.777 | 0.779 | 0.785 | 0.772 | 0.786 | 0.779 | 0.780 | 0.782 | 0.781 | 0.791 |
| K005 | Healthy | 0.726 | 0.717 | 0.716 | 0.720 | 0.717 | 0.716 | 0.719 | 0.714 | 0.710 | 0.731 |
| K014 | Healthy | 0.672 | 0.665 | 0.662 | 0.661 | 0.661 | 0.657 | 0.663 | 0.661 | 0.663 | 0.678 |
| K016 | Healthy | 0.635 | 0.633 | 0.634 | 0.626 | 0.635 | 0.628 | 0.632 | 0.624 | 0.626 | 0.643 |
| K047 | Healthy | 0.756 | 0.756 | 0.756 | 0.757 | 0.755 | 0.755 | 0.756 | 0.753 | 0.751 | 0.761 |
| K054 | Healthy | 0.795 | 0.795 | 0.792 | 0.791 | 0.793 | 0.795 | 0.792 | 0.787 | 0.791 | 0.805 |
| K055 | Healthy | 0.803 | 0.790 | 0.790 | 0.793 | 0.795 | 0.792 | 0.794 | 0.801 | 0.790 | 0.807 |
| K056 | COPD | 0.818 | 0.817 | 0.813 | 0.812 | 0.811 | 0.807 | 0.816 | 0.806 | 0.805 | 0.83 |
| K057 | Healthy | 0.802 | 0.798 | 0.804 | 0.797 | 0.801 | 0.798 | 0.794 | 0.803 | 0.805 | 0.815 |
| K060 | Healthy | 0.794 | 0.794 | 0.790 | 0.789 | 0.795 | 0.792 | 0.794 | 0.798 | 0.795 | 0.81 |
| K072 | COPD | 0.657 | 0.663 | 0.661 | 0.653 | 0.670 | 0.661 | 0.672 | 0.672 | 0.670 | 0.673 |
| K079 | COPD | 0.686 | 0.672 | 0.675 | 0.671 | 0.681 | 0.676 | 0.684 | 0.686 | 0.680 | 0.689 |
| K083 | COPD | 0.738 | 0.737 | 0.736 | 0.729 | 0.731 | 0.737 | 0.727 | 0.736 | 0.736 | 0.746 |
| K084 | COPD | 0.774 | 0.777 | 0.778 | 0.776 | 0.777 | 0.774 | 0.779 | 0.779 | 0.775 | 0.783 |
| K085 | COPD | 0.789 | 0.785 | 0.786 | 0.780 | 0.785 | 0.785 | 0.781 | 0.783 | 0.781 | 0.794 |
| K086 | COPD | 0.656 | 0.658 | 0.661 | 0.671 | 0.672 | 0.674 | 0.658 | 0.666 | 0.658 | 0.685 |
| K147 | Healthy | 0.638 | 0.625 | 0.633 | 0.632 | 0.632 | 0.635 | 0.638 | 0.632 | 0.637 | 0.644 |
| Mantel's R | NA | 0.289 | 0.254 | 0.276 | 0.218 | 0.272 | 0.236 | 0.271 | 0.23 | 0.263 | 0.339 |
| No. of genes | NA | 233 | 235 | 234 | 284 | 238 | 235 | 268 | 243 | 264 | 305 |

**Table S7.** The number of PICRUSt2-inferred pathways that were significantly differentially abundant between COPD and controls (FDR *P*<0.05), based on full-length 16S sequences and sequences from individual hypervariable regions.

| **16S region** | **No. of significant pathways** | **No. of significant pathways supported by metagenome*** | **No. of unique pathways**** | **No. of unique pathways supported by metagenome** |
| --- | --- | --- | --- | --- |
| V1V2 | 43 | 32 | 6 | 2 |
| V1V3 | 56 | 48 | 1 | 0 |
| V1V9 | 57 | 49 | 9 | 8 |
| V2 | 35 | 26 | 1 | 0 |
| V3 | 37 | 23 | 6 | 2 |
| V3V4 | 40 | 32 | 3 | 2 |
| V3V5 | 47 | 36 | 1 | 1 |
| V4 | 57 | 31 | 5 | 0 |
| V6V8 | 49 | 36 | 2 | 2 |
| V6V9 | 52 | 31 | 4 | 0 |

* The pathways that had same direction of changes based on PICRUSt2 inference and in the actual metagenome.

** The pathways that were statistically significant in the inference based on the specific 16S region but non-significant based on any other regions.

**Table S8.** The list of 9 PICRUSt-predicted pathways uniquely identified as significantly different in abundance between COPD and healthy controls by the full-length 16S data. For each pathway, their abundances, log2 fold-changes (FC) and FDR *P*-values were shown for the full-length (V1V9) data and sub-region data as average values. All these pathways were statistically significant in V1V9 data but non-significant in any of the nine sub-region data. Also shown were their corresponding FC and FDR *P*-values in the metagenomic data of the subset 10 COPD and 10 healthy samples.

| **Pathway ID** | **V1V9_abundance** | **V1V9_FC** | **V1V9_FDR** | **Average_abundance** | **Average FC** | **Average FDR** | **metagenome_FC** | **metagenome_FDR** | **Description** |
| --- | --- | --- | --- | --- | --- | --- | --- | --- | --- |
| PWY-5676 | 289.872 | -0.661 | 0.004 | 131.514 | -0.174 | 0.266 | -1.608 | 0.248 | acetyl-CoA fermentation to butyrate |
| PWY-5484 | 1347.833 | -0.399 | 0.007 | 718.903 | -0.121 | 0.304 | -1.271 | 0.306 | glycolysis II (from fructose 6-phosphate) |
| PWY490-3 | 256.695 | -0.544 | 0.028 | 107.023 | -0.112 | 0.219 | -2.502 | 0.168 | nitrate reduction |
| PWY-7219 | 1938.478 | -0.276 | 0.03 | 1006.231 | -0.106 | 0.396 | -1.363 | 0.315 | adenosine ribonucleotides de novo biosynthesis |
| GLYOXYLATE-BYPASS | 129.744 | 1.042 | 0.032 | 100.389 | 0.524 | 0.238 | -0.992 | 0.405 | glyoxylate cycle |
| THRESYN-PWY | 1550.343 | -0.26 | 0.035 | 800.959 | -0.096 | 0.446 | -1.103 | 0.435 | superpathway of L-threonine biosynthesis |
| PWY-6386 | 1741.622 | -0.274 | 0.037 | 897.568 | -0.105 | 0.397 | -1.448 | 0.315 | UDP-N-acetylmuramoyl-pentapeptide biosynthesis II |
| PWY-5695 | 1452.145 | -0.259 | 0.038 | 785.254 | -0.116 | 0.292 | -1.482 | 0.436 | urate biosynthesis/inosine 5'-phosphate degradation |
| PWY-6123 | 1790.831 | -0.183 | 0.041 | 922.248 | -0.114 | 0.32 | -1.013 | 0.439 | inosine-5'-phosphate biosynthesis I |

**Table S9.** The average and standard deviation of Ct values for butyryl-CoA:acetate CoA-transferase gene (EC:2.8.3.8) and internal control (*rpoB* gene) in a subset of 87 COPD and healthy subjects. The values for ΔCt, ΔΔCt, 2^-ΔΔCt (COPD vs Healthy) and decrease fold change are shown.

|  | **EC:2.8.3.8** | **rpoB gene** |
| --- | --- | --- |
| COPD (Ct) | 33.17±3.36 | 25.97±3.03 |
| Healthy (Ct) | 31.18±3.43 | 26.09±3.05 |
| COPD (ΔCt) | 8.20±3.00 |  |
| Healthy (ΔCt) | 6.09±2.24 |  |
| ΔΔCt (COPD vs Healthy) | 2.105 |  |
| 2^-ΔΔCt | 0.232 |  |
| Decrease folds | 4.317 |  |
